# Chemical Ecology of *Capnodis tenebrionis* (L.) (Coleoptera: Buprestidae): Behavioral and Biochemical Strategies for Intraspecific and Host Interactions

**DOI:** 10.3389/fphys.2019.00604

**Published:** 2019-05-27

**Authors:** Giuseppe Bari, Andrea Scala, Vita Garzone, Rosanna Salvia, Cem Yalcin, Pasqua Vernile, Antonella Maria Aresta, Osvaldo Facini, Rita Baraldi, Sabino A. Bufo, Heiko Vogel, Enrico de Lillo, Francesca Rapparini, Patrizia Falabella

**Affiliations:** ^1^Department of Soil, Plant and Food Sciences, University of Bari Aldo Moro, Bari, Italy; ^2^Department of Science, University of Basilicata, Potenza, Italy; ^3^Syngenta, Izmir, Turkey; ^4^Department of Chemistry, University of Bari Aldo Moro, Bari, Italy; ^5^Department of Biology, Agriculture and Food Sciences, Biometeorology Institute, National Research Council, Bologna, Italy; ^6^Department of Entomology, Max Planck Institute for Chemical Ecology, Jena, Germany

**Keywords:** chemoreception, mating, Mediterranean flat-headed root-borer, scanning electron microscopy, soluble olfactory proteins, volatile organic compounds

## Abstract

This study focuses on several aspects of communication strategies adopted by adults of the Mediterranean flat-headed root-borer *Capnodis tenebrionis* (Coleoptera: Buprestidae). Morphological studies on the structures involved in mate recognition and acceptance revealed the presence of porous areas in the pronota in both sexes. These areas were variable in shape and size, but proportionally larger in males. The presence of chaetic, basiconic, and coeloconic sensilla in the antennae of both males and females was verified. Bioassays revealed stereotyped rituals in males and the involvement of female pronotal secretions in mate recognition and acceptance. During the mating assays, the female’s pronotum was covered by a biologically inert polymeric resin (DenFil^TM^), which prevented males from detecting the secretions and from completing the copulation ritual. The use of the resin allowed for the collection of chemical compounds. GC-MS analysis of the resin suggested it may be used to retain compounds from insect body surfaces and revealed sex-specific chemical profiles in the cuticles. Since adult *C. tenebrionis* may use volatile organic compounds (VOCs) emitted from leaves or shoots, the VOC emission profiles of apricot trees were characterized. Several volatiles related to plant-insect interactions involving fruit tree species of the Rosaceae family and buprestid beetles were identified. To improve understanding of how VOCs are perceived, candidate soluble olfactory proteins involved in chemoreception (odorant-binding proteins and chemosensory proteins) were identified using tissue and sex-specific RNA-seq data. The implications for chemical identification, physiological and ecological functions in intraspecific communication and insect–host interactions are discussed and potential applications for monitoring presented.

## Introduction

*Capnodis tenebrionis* (L.) (Coleoptera: Buprestidae), commonly known as the Mediterranean flat-headed root-borer, affects many species of Rosaceae, particularly apricot, peach, plum, nectarine, cherry, and almond (Malagon et al., 1990; Ben-Yehuda et al., 2000; Marannino and de Lillo, 2007; Morton and García del Pino, 2008). The beetle is common in Central and Southern Europe, Northern Africa and the Middle East (Garrido et al., 1987; Tezcan, 1995; Martin et al., 1998; Cınar et al., 2004; Marannino and de Lillo, 2007; Sharon et al., 2010). *Capnodis tenebrionis* can be a key pest in some areas and cultivation conditions, particularly in organic orchards and/or in arid and semiarid environments, where plants are susceptible to the destructive action of larvae on roots and control strategies have to be applied (Bonsignore and Bellamy, 2007; del Mar Martinez de Altube et al., 2008). Recent outbreaks in areas like Emilia Romagna and Southern France, previously less affected by this pest, may be a consequence of global warming, especially for trees growing on clayey and poorly irrigated soils, and may be related to the beetle preference for the high temperature (Bonsignore and Vacante, 2009).

Adult beetles feed on the bark of shoots, buds, and leaf petioles, and usually prefer weakened and diseased trees rather than vigorous ones (Rivnay, 1946; Garrido, 1984; Bonsignore and Bellamy, 2007). These adults can seriously damage young trees in nurseries, orchards and greenhouses, but rarely affect established, well-cultivated and irrigated fruit-bearing orchards (García del Pino and Morton, 2005). Females lay eggs in the cracks of dry soil or under stones, close to trees and rarely on the bark (Ben-Yehuda et al., 2000). Neonate larvae crawl within the soil, penetrate the roots and feed on the root cortical and subcortical tissues (Mendel et al., 2003). Damage caused by larvae becomes obvious as the tree dries out or begins to secrete resin (Marannino et al., 2004; del Mar Martinez de Altube et al., 2008). One-year-old trees can be killed by a single larva; a few larvae can lead to the death of a mature tree within 1 or 2 years (Ben-Yehuda et al., 2000; García del Pino and Morton, 2005).

Whether *C. tenebrionis* secretes volatiles – long-distance airborne sex or aggregation pheromones – is not yet known (Bari et al., 2004; Rodriguez-Saona et al., 2006; Sharon et al., 2010), and how beetles find, select and accept partners has yet to be described. However, male-biased aggregation on host trees may be linked to mating (Bonsignore et al., 2008; Bonsignore and Jones, 2013). Additionally, cuticular hydrocarbons, which have been discovered in another buprestid, *Agrilus planipennis* (F.), may function as contact pheromones and be involved in the beetle’s mating behavior (Lelito et al., 2009; Silk et al., 2009). Plant-produced chemicals could be involved in host location by *C. tenebrionis* (Sharon et al., 2010). It is well known that volatile organic compounds (VOCs) released by plants provide olfactory cues to help herbivorous insects locate nutritional resources and suitable oviposition sites, within both natural and agricultural ecosystems (Bruce et al., 2005, and references therein). VOC emissions from healthy plants result in a species-specific pattern that can be altered by abiotic and biotic factors (Niinemets et al., 2013). Emissions of VOCs from both healthy and stressed plants have been suggested to explain the host preference behavior of adult *C. tenebrionis* when testing the effect of air headspace of apricot twigs in an olfactometer assay (Sharon et al., 2010). Although species-by-species surveys of VOCs emissions have been carried out for forested ecosystems, VOC emissions from agricultural species, especially fruit trees, remain poorly characterized (Staudt et al., 2010; Najar-Rodriguez et al., 2013).

Since adult mating behavior likely involves the emission and recognition of pheromone cues, studying these behaviors may suggest the putative role of the structures involved in their release and perception. Similarly, knowledge of which volatiles are released by trees may clarify how adult beetles choose trees for feeding or mating. Both sets of data could improve monitoring strategies of *C. tenebrionis* in the field. Antennae in insects are the typical organs involved in chemical perception; a chemical message (host plant volatiles, pheromones or allomones) is transduced into a neuronal impulse starting from the olfactory sensory neuron (Jacquin-Joly and Merlin, 2004; Leal, 2013). Olfactory, gustatory, and ionotropic receptors, soluble olfactory proteins such as odorant-binding proteins (OBPs) and chemosensory proteins (CSPs) mediate chemical perception in insects (Fan et al., 2010; Mamidala et al., 2013). There is still no consensus about the role of each olfactory gene family in Coleoptera and few studies investigated this issue in coleopteran insects (Engsontia et al., 2008; Mitchell et al., 2012; Andersson et al., 2013). Concerning buprestids, Mamidala et al. (2013) focused on gene families associated with odor processing and xenobiotic degradation in the invasive *A. planipennis*. A detailed study of the distribution and structure of *A. planipennis* antennal sensilla showed that males had significantly more uniporous gustatory sensilla than females (Crook et al., 2008), supporting the hypothesis that mate recognition by males involves female-released contact cues. The antennal morphology and the sensillar arrangement of *C. tenebrionis* adults are still poorly known, as are the antennal pathways that allow for volatile or non-volatile compound binding, transport and olfactory neuron responses.

To understand the chemical ecology of *C. tenebrionis*, an investigation was carried out with the following goals: (1) to characterize the behavior and the structures involved in mate recognition and acceptance, (2) to identify the types of cues involved in mate recognition, (3) to describe the antennal sensillar morphology in males and females, (4) to characterize the qualitative pattern of constitutive VOC emissions by the host plant under non-stressed conditions and determine how these emissions act as infochemicals in host recognition and preference, (5) to recognize and evaluate the expression level of OBPs and CSPs, proteins involved in chemoreception, on the basis of the functional annotation and the analysis of conserved amino acid patterns. The identification and the evaluation of OBPs and CSPs expression level was performed starting from the transcriptomic analysis of antennae, the main organs involved in chemoreception in which soluble olfactory proteins are commonly highly expressed, in comparison to the level of the expression of the same proteins in the rest of the insect body.

## Materials and Methods

### Source of Adult Beetles

Due to the prolonged life cycle of *C. tenebrionis* and the difficulties in the rearing of the insect in laboratory and artificial conditions, the present study was carried out from 2011 to 2017, and on adults collected from infested orchards of apricot, cherry, peach, and plum in the District of Bari and Matera (Southern Italy). Adult beetles were collected from March to October from the host plants by hand or with a net. Active, healthy adults were held in ventilated polystyrene (17 cm × 25 cm × 7 cm) (de Lillo, 1998) or metal net cages (30 cm × 30 cm × 30 cm) (5–15 beetles per cage). Beetles were maintained under controlled conditions (28 ± 1°C, 45 ± 5%RH, 16:8 L:D photoperiod) or at room temperature, depending on the assay and observations to be performed. Insects were fed with fresh apricot and plum twigs. Cages were inspected every 2–7 days in order to renew twigs, and to remove feces and dead adults.

Females laid eggs on cellulose discs; eggs were incubated at 27 ± 1°C in darkness for 8–15 days until neonates hatched. Twenty-four-hour-old neonates were transferred with a fine brush onto Petri dishes (Ø 5 cm) containing artificial diet (Gindin et al., 2009). Diet included root cortex flour of 2- to 3-year-old Myrobalan (*Prunus cerasifera* L.) trees. Diet was replaced every 2 weeks up to pupation. Larvae were reared in a dark chamber with controlled temperature at 27 ± 1°C. Cuticular extractions were performed on newly emerged (virgin) adults.

### Light Microscopy Examination of the Pronotum

Ten males and 10 females were randomly selected from a large collection of dead specimens and their pronota were removed under a dissecting stereomicroscope (Weatler, Ernst Leitz, Wetzlar, Germany). The pronotum of each specimen was mapped and pencil-drawn at 20X through a camera lucida mounted on a dissecting stereomicroscope (SZH, Olympus, Tokyo, Japan). Particular care was given to the details of the pronotum surface covered with a whitish dust (fields of secretion). The drawings were digitized (Perfection 3200 Photo, Epson, Nagano, Japan), and the smooth and rough surface fields of each specimen were measured (in pixels) using a graphic editing software (Adobe^®^Photoshop^®^CS5 Extended). The coverage percentage of smooth and rough fields was compared to the whole surface of the pronotum for both genders. Non-normally distributed continuous data were analyzed using the non-parametric Mann–Whitney *U* test.

### SEM Examination of the Adult Pronotum and Antennae

Pronota were dissected from six males and six females, randomly selected from a large collection of dead specimens. The outer and inner surfaces were cleaned by washing the pronota in a solution composed of (1) ethylene 95% and xylene (1:1) (Sands, 1984) for 10 min (for three males and three females) or (2) sodium lauryl sulfate (25 g), sodium hydroxide (25 g), liquid soap (25 g) and distilled water (500 mL) (for three males and three females) (Porcelli and Di Palma, 2001) for a few days, followed by a treatment with Essig’s fluid for a further day (Wilkey, 1962).

After three females and three males were frozen, their pairs of antennae were cut at the base of the scape and shaken for 24 h (15 shakes per min) in a solution containing a denture-cleaning tablet (Polident^®^, Brentford, Middlesex, United Kingdom) (1.2 g/100 mL of distilled water). Finally, antennae were washed in distilled water.

Cleaned pronota were air-dried at room temperature and antennae were dried in a dehydrator. Both structures were platinum-coated using a sputter coater (S150A, Edwards High Vacuum International, Crawley, United Kingdom) and examined under a scanning electron microscope (SEM) (Tabletop Microscope 3000, Hitachi, Tokyo, Japan) at 5 kV of accelerating voltage. At the same time, antennae of other three frozen females and three frozen males were not treated with Polident but directly platinum-coated and examined under SEM. The results (not shown) were compared with the antennae treated with Polident. Surfaces and sensilla were largely covered by adhering dusts and particles in antennae not treated with Polident, whereas they were clean and well exposed when antennae were treated with Polident. No damages of these structures were observed.

### SEM Examination of the Pronotal Resin Copy

**Figure 1:**
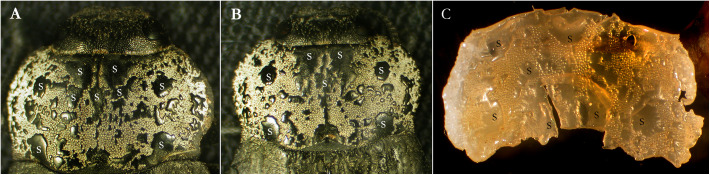
Pronota of *C. tenebrionis*: smooth fields and rough fields on the pronotum of the adult female **(A)** and male **(B)**. Resin copy **(C)** removed from the pronotum. S, smooth fields; R, rough fields.

Males and females were collected in early spring after their overwintering. They were reared as previously described (see section “Source of Adult Beetles”) and were assayed at the appearance of a white waxy secretion on the pronotum. These adults were assayed in a Y-shaped olfactometer (Bari et al., 2004) and females able to attract males and males moved toward the arm baited by females were used for subsequent observations. The pronota of these live beetles were coated with a polymeric resin-based dental restorative material (DenFil^TM^, Vericom Ltd., Co., Gyeonggi-do, South Korea). This creamy composite is silicon-like at room temperature, hardens under ultraviolet light (UV) within about 20 s (or under light source for longer time) and contains barium aluminosilicate (average particle size ≥ 1 μm), fumed silica (average particle size 0.04 μm), bisphenol A glycidyl-dimethacrylate, triethylene-glycol dimethacrylate and other trace compounds. The resin was applied using forceps with flattened tips to cover the whole pronotum area including its lateral margins. After the resin hardened under a white fluorescent light, it was lifted gently and removed. Resin was applied on both male and female pronota. After the resin was removed from the beetle’s surface, a resin copy of the pronotum with its secretions and the most external layers was made (Figure 1). Pure resin material was hardened and used as a blank (negative) control. Resin copy was studied under an SEM (Tabletop microscope 3000, Hitachi, Tokyo, Japan), at 5 kV of accelerating voltage. The chemical spectrum of the beetle-covering compounds of the pronotum adhering to the resin was analyzed using an energy dispersive spectrometer (EDS) on surface spots.

### Pre-mating and Mating Behavior

Although the age and mating status of field-caught beetles were unknown, adults caught in early spring (March-April 2011) were presumed to be sexually immature. Some females (about a dozen) were dissected after collection and sperms were not found in their spermatechae. The beetles were reared as previously described (see section “Source of Adult Beetles”), until the appearance of the white waxy secretion on the pronotum. Males and females were assayed in a Y-shaped olfactometer (Bari et al., 2004), and only females able to attract males and males moved toward the arm baited by females were used for the mating bioassay. Males (*n* = 22) and females (*n* = 25) were coupled in mating arenas (15 cm × 34 cm × 6 cm) at 28 ± 1°C under fluorescent light. The assay was repeated over several consecutive days. Each observation (*n* = 200) lasted 30 min at most and the behavior of each individual was studied before, during and shortly after copulation. Coupling duration was also recorded. Males were kept separate from females for at least 12 h before the assay. Coupling behavior was repeatedly observed for the same individuals, in order to determine whether females and males mated once or more than once under the experimental conditions. The outline of the beetle’s body was approximated to an ellipse. Therefore, the body length (from the head tip to the tip of the elytra end) and width of the pronotum of mated couples were measured with a caliper; the surface of this ellipse was calculated. Pearson’s bivariate analysis and Student’s *t*-test (*n* = 19, *p* < 0.05, two-tailed) were performed in order to verify the relationship between mating duration and body size of mated couples.

Assuming that sexually mature males could ascertain female maturity via a contact pheromone released on the pronotum’s secretory area, soon after the selection of adults by the olfactometer assays, the following procedure was carried out: (1) Ten males and 10 females (the most active following the olfactometer assay) were placed in mating arenas (glass container, 15 cm × 34 cm × 6 cm in size, with bottom and lateral sides covered by white paper). Once individual’s readiness to mate was verified, they were separated until just before copulation. (2) Afterward, the lateral half (right or left) of the pronota of five out of ten selected females was coated with Denfil in order to evaluate the repellency of the resin on males (this assay was considered a sort of control). After the resin hardened, couples were again placed together and the males’ behavior was observed for up to 30 min. This assay was repeated five times with different male-female combinations. (3) Next, the uncoated half of the female’s pronotum was also treated by resin to hide the whole pronotum from the male’s antennae; couples were formed again and the male’s behavior was observed for up to 30 min. In addition, five additional couples were formed and observed, in which each female began with a whole uncoated pronotum that was then covered entirely. (4) Finally, the resin was removed from the pronota of all females, couples (*n* = 10) were again paired and individuals were left to mate.

### Chemical Analysis of Pronotum Secretions and the Whole Beetle Body

The pronota of adults showing white wax secretion were used. The hardened resin acted like a negative, revealing the pronotum secretions and outer cuticular layers. After the resin copies were removed, they were dipped in a solution of 0.3 mL of *n*-hexane and sonicated for 20 min in an ultrasonic bath. A pure polymerized resin was used as a blank. In 2014, resin copies of the pronota were obtained from males and females caught in the field in early spring (after their overwintering) and selected from among those which positively responded to the Y-olfactometer and mating assays. In 2015, resin copies were produced by three groups: virgin males and females obtained by rearing larvae (without overwintering); mated males and females caught in the field (after their overwintering); adults caught in the field (after their overwintering) which were demonstrated to be attracted (males) and attractive (females) by the olfactometer bioassay. In 2015, cuticular chemicals were also directly extracted from the whole bodies of virgin males and females (without overwintering) whose larvae had been reared on artificial diets in order to highlight further chemicals potentially emitted from other body territories. Beetles used in the preparation of the whole body extracts were the most vigorous and active in cages. They were starved for 24 h in order to avoid contamination with food or feces during the extraction. Specimens were freeze-killed and left to dry at room temperature for 20 min. They were individually dipped in 3 mL aliquots of *n*-hexane for 15 min each. After removing the whole bodies, extracts were sonicated for 20 min in an ultrasonic bath. Three specimens were used as replicas for each year, gender, and treatment (resin copy of the pronotum, whole body, field-caught adults, virgin adults).

In all cases, the *n*-hexane was concentrated in a gentle stream of nitrogen to 0.3 mL and stored at -20°C till the analysis (Spikes et al., 2010). Of the extracted solution, 1–3 μL was injected in the GC-MS^n^ system (GCMSD 5975C, Thermo Electron Corporation, Austin, TX, United States) composed by a TRACE ultragas chromatograph (GC) interfaced to a Finnigan PolarisQ ion trap mass spectrometer (MS). The GC was provided by a capillary column made by HP-5MS (30 m length, 0.25 mm I.D. with 0.25 mm film thickness, 5 inch cage, Agilent, Palo Alto, CA, United States). The applied GC conditions were as follows: injector temperature (splitless mode), 200°C; carrier gas (helium) flow rate, 0.8 mL/min; the oven temperature program was raised from an initial 50°C (1 min) to 240°C at 10°C/min and 20°C/min up to 300°C (5 min) (Ginzel et al., 2006); transfer line temperature was 280°C. The applied MS conditions were as follows: EI^+^ operating mode; source temperature, 250°C; electron energy, 70 eV; current, 200 μA at the filament; spectra acquisition (total ion current, TIC), from 50 to 650 m/z. To better identify the cuticular compounds of the whole beetle body, 3 μL of a C7–C40 saturated alkane standard (STD) Supelco 49452U was injected under the same conditions.

Compounds were identified based on their MS spectra (NIST05, WILEY Masslib) and the obtained peak values for STD. The differences between male, female and control (pure resin) chromatograms were evaluated.

### Plant VOCs: Collection and Analysis

Volatile compounds were collected from the apricot (*Prunus armeniaca* L.) cultivar FARBALY^®^, which was selected because it represents a widely used cultivar, often attacked by *C. tenebrionis*. Three-year-old potted plants were grown in well-watered universal potting soil in the nursery of IBIMET-CNR in Bologna, Italy, under natural conditions of light, temperature, and humidity. VOC emissions were taken from three healthy leaves of non-fruit-bearing branches of four plants during fruit ripening (July–August 2015). The physiological status of the leaves was monitored through measurements of carbon assimilation, stomatal conductance and transpiration rates using the LI-COR 6400 Photosynthesis System (LI-COR Biosciences Inc., Lincoln, NE, United States) by placing each fully developed leaf in a 6 cm^2^ cuvette. Measurements were performed at reference CO_2_ (400 μmol mol^-1^), flow rate (500 μmol min^-1^), photosynthetic active radiation (PAR) (1000 μmol m^-2^ s^-1^) and at 30°C with 30–50% of relative humidity. Carbon assimilation was measured for approximately 2–4 min, until photosynthetic intensity stabilized. VOC emissions were analyzed simultaneously with gas exchange measurements. Air samples were taken from the cuvette using steel tubes packed with 200 mg of Tenax GC^®^and Carbograph (Markes International, Ltd., Llantrisant, United Kingdom) connected to an external pump (Pocket Pump SKC Inc., Eighty Four, PA, United States), adsorbing at a flow rate of 200 mL min^-1^ for 30 min. All the adsorbent tubes were kept at -20°C until analysis to avoid any chemical alteration and/or artifacts. Traps were analyzed using a thermal-desorber (Markes International, Series 2 Unity) connected to a 7890A gas chromatograph coupled with a 5975C mass detector (GC-MS, Agilent Technologies, Wilmington, DE, United States) (Baraldi et al., 2018). Identification of the sampled compounds was carried out by comparing their retention times and mass spectra with those of authentic standards (Rapparini et al., 2004).

### Olfactometer Assays With Plant VOCs

Adult beetles were assayed during 2013 using a transparent glass Y-olfactometer (55.6 mm internal diameter) composed of a common stem (about 15 cm long) bearing two lateral arms (about 10 cm long) separated at a 75° angle on the horizontal plane. The inner bottom of the tube housed a strip of fine metal netting to provide traction for the beetle. The olfactometer was placed horizontally and was illuminated by a white fluorescent light tube (700 lux), mounted at about 1 m above the olfactometer. The assay was carried out using cis-3-hexen-1-ol 98.0% (Aldrich), benzaldehyde 99.0% (Fluka), 2-hexanone reagent grade 98.0% (Sigma-Aldrich), 1-pentanol ≥ 99.0% Sigma-Aldrich), 3-methyl-1-butanol reagent grade (Sigma-Aldrich), S-(-)-limonene analytical standard (Fluka), β-myrcene analytical standard (Fluka) as volatile compounds, at a concentration of 1M in HPLC grade *n*-hexane. These compounds were selected based on the results of a previous electro-antennographic study of the response of *C. tenebrionis* adults to some chemicals (Bari et al., 2004), and VOC emissions of *Prunus* species (Baraldi et al., 1999). Each volatile compound was tested on 15 males and 15 females selected among the most active adults caught in the field during spring and reared under controlled conditions for at least 1 month. A 0.5 cm^2^ square piece of filter paper (Whatman^TM^, Maidstone, Kent, United Kingdom) was added with 5 μL of the volatile compound solution and introduced in the terminal glass bulb of one arm (the baited arm). Pure solvent was introduced on the glass bulb of the other arm (the control arm). Air passed through an activated charcoal filter and distilled water in a 1 L flask, and continued through flowmeters into the arms at an airstream of 0.9 L min^-1^ arm^-1^. A single adult was introduced into the stem and allowed to move freely toward the arms and against the air flow. Each test lasted 15 min and arm choice was recorded. Responses were classified as “no-choice” when adults did not select an olfactometer arm within 15 min. After five adults had been assayed, the olfactometer arms were flipped 180° to minimize directional bias. The assay was carried out in a dark room, lighted only by the fluorescent tube above the olfactometer, and at 28 ± 1°C. At the end of the observations with the same volatile compound, the olfactometer was rinsed with soapy water and analytical grade acetone, and air-dried. After each assay, the adults were returned to the cages as above and used for the next assays. Each assayed adult was considered a replica. Results were expressed as response index calculated as:

$$\mathrm{number}\;\mathrm{of}\;\mathrm{adults}\;\mathrm{moved}\;\mathrm{to}\;\mathrm{the}\;\mathrm{baited}\;\;=\;\frac{\mathrm{arm}\;-\;\mathrm{number}\;\mathrm{of}\;\mathrm{adults}\;\mathrm{moved}\;\mathrm{to}\;\mathrm{non}-\mathrm{baited}\;\mathrm{arm}}{\mathrm{total}\;\mathrm{number}\;\mathrm{of}\;\mathrm{assayed}\;\mathrm{adults}\;\mathrm{per}\;\mathrm{each}\;\mathrm{tested}\;\mathrm{VOC}}\;\times \;100\;\;\;\;\;\;\;\;\;\;\;\;\;\;\;\;\;(1)$$

### RNA Extraction and cDNA Synthesis

Antennae from 30 females and 30 males, caught in the field in early spring (after their overwintering), were cut from the base of the scape of live insects; a single male body and a single female body, both without antennae, were collected. Samples were frozen in liquid nitrogen, homogenized in centrifuge tubes containing TRI Reagent (Sigma, St. Louis, MO, United States) and stored at -80°C until RNA extraction. Total RNA was extracted using TRI Reagent following the manufacturer’s instructions (Sigma, St. Louis, MO, United States). A DNase (Turbo DNase, Ambion Inc., Austin, TX, United States) treatment was carried out to eliminate any contaminating DNA. After DNase enzyme removal, RNA was further purified using the RNeasy MinElute Cleanup Kit (Qiagen, Venlo, Netherlands) following the manufacturer’s protocol and eluted in 20 μL of RNA storage solution (Ambion Inc., Austin, TX, United States). RNA integrity was verified on an Agilent 2100 Bioanalyzer using RNA nano chips (Agilent Technologies, Palo Alto, CA), while RNA quantity was determined by a NanoDrop ND1000 spectrophotometer (Thermo Scientific, Waltham, MA, United States).

### Sequencing and *de novo* Transcriptome Assembly

Transcriptome sequencing of all RNA samples was performed with poly(A)+ enriched mRNA fragmented to an average of 150 nucleotides. Sequencing was carried out by the Max Planck Genome Center^1^ using standard TruSeq procedures on an Illumina HiSeq2500 sequencer. Quality control measures, including the filtering of high-quality reads based on the score given in FASTQ files, the removal of reads containing primer/adaptor sequences and the trimming of read lengths, were carried out using CLC Genomics Workbench v9.1. The *de novo* transcriptome assembly was carried out using the same software, which is designed to assemble large transcriptomes using sequences from short-read sequencing platforms. All obtained sequences (contigs) were used for BLASTX searches (Altschul et al., 1997) against the National Center for Biotechnology Information (NCBI) non-redundant (nr) database, considering all hits with an e-value < 1E-5. The transcriptome was annotated using BLAST, gene ontology and InterProScan searches using BLAST2GO PRO v4.1^2^ (Götz et al., 2008). To optimize annotation of the obtained data, we used GO slim, a subset of GO terms that provides a high level of annotations and allows a global view of the result.

### Digital Gene Expression Analysis

The tblastn program was used, with available sequences of OBP and CSP proteins from different insect species, as a “query” to identify candidate unigenes encoding putative OBPs and CSPs in *C. tenebrionis* adult males and females. All candidate proteins were manually checked by the blastx program at the NCBI.

The abundance in the expression level of each “unique” nucleotide sequences (contigs) was calculated on the basis of the reads per kilobase per million mapped reads method (RPKM) (Mortazavi et al., 2008), following the formula:

RPKM(A) = (10, 00, 000 × C × 1000)/(N × L)

where RPKM (A) is the abundance of gene A, *C* is the number of reads that uniquely align to gene A, *N* is the total number of reads that uniquely align to all genes, and *L* is the number of bases in gene A.

## Results

### Light Microscopy Examination of the Pronotum

Smooth and rough fields were observed on both antimers of the adult pronota (Figure 1). The pronotum is provided with one pre-scutellar dimple, shaped like a horseshoe, on the posterior margin. Two pairs of almost rounded and prominent smooth fields are found on the lateral sides. Size and distribution of smooth and rough fields, in both males and females, are not perfectly symmetrical and were not perfectly the same (for size and position) in all studied specimens. In addition, the borders of these areas appear quite irregular.

The average percentage of the surface size of the rough fields in comparison to the whole pronotum surface was not statistically different between the two genders.

### SEM Examination of the Adult Pronotum and Antennae

**Figure 2:**
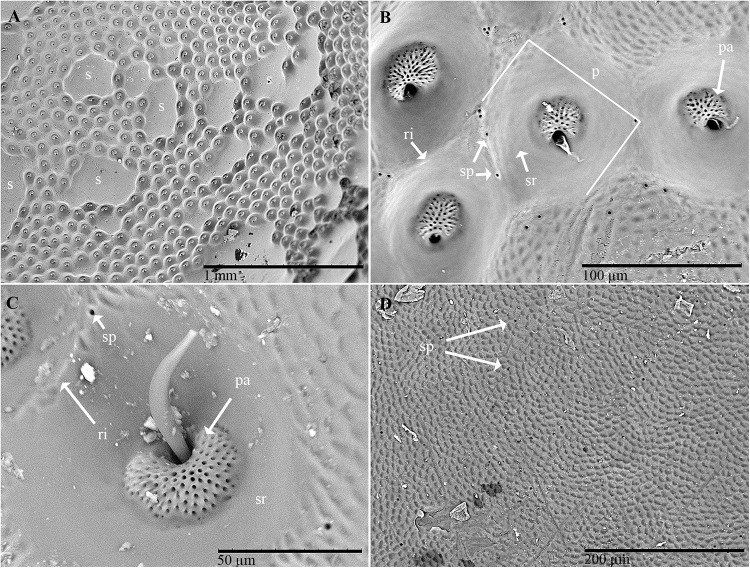
Scanning electron micrographs of *C. tenebrionis*: general view **(A)** and details of a few glandular openings **(B)** on rough fields of the male’s pronotum; **(C)** glandular openings on the male’s pronotum; **(D)** outer surface of the female’s pronotum. Specimens in panels **(A,B,D)** were washed according to Porcelli and Di Palma (2001); the specimen in panel **(C)** was washed according to Sands (1984). s, smooth fields; p, pore pit complex; pa, pore pit area; r, rim; sp, single pore; sr, smooth ring.

Examination of the outer surface of the pronotum by SEM revealed the presence of groups of slight depressions (“pore pits”) on the rough fields (Figure 2A–C). Most of the pore pits were hexagonal or round (Figure 2A,B), but some were irregularly outlined. The center of each complex was depressed and exhibited a cribrous (perforated) circular- or ovoid-shaped area, 50–60 μm in diameter, composed of 60–80 small circular or ovoid pores (each 5–10 μm in diameter) and one large pore (20–30 μm in diameter) (Figure 2B,C). In many cases, waxy filaments were observed to protrude from the pores (Figure 2B,C). The cribrous area was surrounded by a smooth ring delimited by a slight rim (Figure 2B,C). The diameter of the whole pore pit complex was between 80 and 120 μm at the rim level. Differences were observed among the specimens due to the cleaning procedure used. Short filaments (Figure 2C) of different thicknesses were detected protruding from the treated pores, according to Sands (1984). No filaments or filament residues were observed on the treated pronota, according to Porcelli and Di Palma (2001) (Figure 2B). Many smallish pores randomly scattered in varying densities on smooth and rough fields, often on the rims of pits, were also detected (Figure 2D). These pores consisted of a circular hole, ranging from 1 to 5 μm in diameter, with a slight border (Figure 2B).

**Figure 3:**
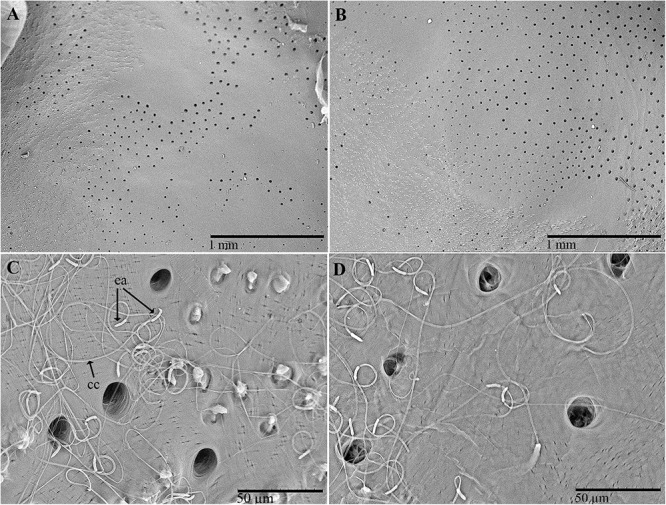
Scanning electron micrographs of *C. tenebrionis*: glandular openings on the inner surface of the female **(A,C)** and male pronota **(B,D)**, washed according to Porcelli and Di Palma (2001). ea, end apparatus; cc, conducting canal.

Several groups of pores (from 10 to 20 μm in diameter) were observed on the inner surface of the pronotum (Figure 3A–D). The overall distribution of these grouped pores appeared to correspond roughly to the distribution of the pore pits of the rough fields on the outer surface. An abundance of filamentous cuticular gland structures was connected to each of these pores and covered the inner surface of the cuticle (Figure 3C,D). The structures were 150–200 μm long and uniform in diameter. The far end of the structure (measured from the pore pit, or the receiving canal) was thicker and spindle-shaped than the filament (also known as the conducting canal) that entered the pore.

No particular differences were detected between the outer and inner surfaces of the pronota belonging to males and those belonging to females.

**Figure 4:**
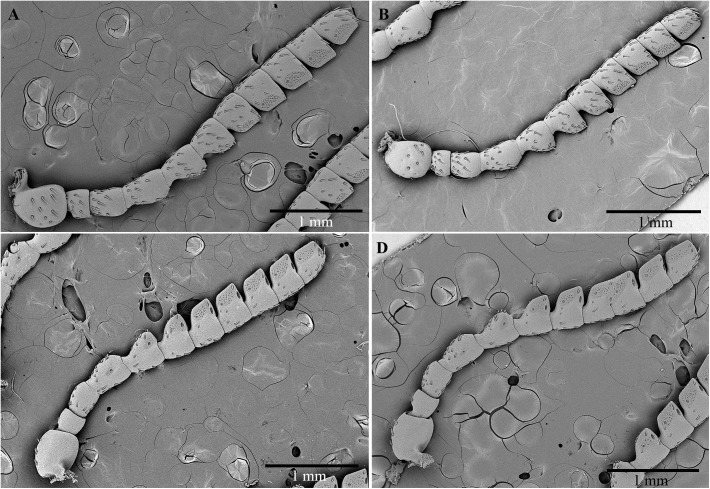
Scanning electron micrographs of antennae of *C. tenebrionis*: general view of the antiaxial side of a female **(A)** and a male **(B)**; paraxial side of a female **(C)** and a male **(D)**.

The size and shape of the antennae in males and females are very similar. No relevant morphometric differences between genders were detected. The antennae are serrate-truncate, composed of 11 articles, long 4.7 ± 0.7 (SD) mm in males (*n* = 3), and 4.6 ± 0.8 mm (*n* = 3) in females (Figure 4). The cross-section is almost rounded for articles I–IV, whereas it appears triangular for articles V–IX. The scape is subglobose and larger than the other articles (591.7 ± 35.1 μm in females and 566.7 ± 52.5 μm in males). The pedicel is subcylindrical, short and stocky, and seems to be longer in females (259.3 ± 20.6 μm) than in males (218.7 ± 26.1 μm). Both articles III and IV have the shape of a barrel, whereas articles V–XI are wedge-shaped.

**Figure 5:**
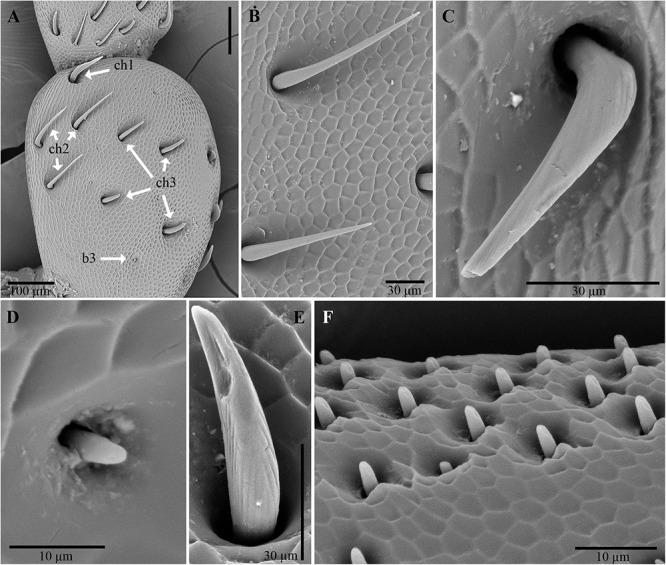
Scanning electron micrographs of antennae of *C. tenebrionis*: antiaxial view of the scape of a male **(A)**; chaetic sensilla subtype I **(B)**, subtype II **(C)** and III **(E)**, and a basiconic sensillum subtype III **(D)** on the antiaxial side of a scape of a female; basiconic sensillum subtype II **(F)** on the antiaxial side of the article IX of a female. b3, basiconic sensillum subtype III; ch1, chaetic sensillum subtype I; ch2, chaetic sensillum subtype II; ch3, chaetic sensillum subtype III.

Male and female antennae are provided with three types of sensilla: chaetic, basiconic, and coeloconic (Altner and Prillinger, 1980; Zacharuk, 1980). The chaetic sensilla are present along the whole antenna and vary in length (38–109 μm). They are similarly distributed between males and females, are more or less evenly distributed on articles I–IV and are concentrated on the dorsal and subdorsal sides of the more distal articles (Figure 5A). The number of chaetic sensilla decreased from articles VI to XI. These sensilla have an apparently rigid shaft that sinks into a socket; there is no rim. This shaft usually shows longitudinal grooves with no evidence of pores, a type of external morphology that is inferred to have a mechanoreceptive function.

Three subtypes of chaetic sensilla can be distinguished on the base of the shaft (Figure 5B,C,E); each is inserted into an individual socket that is without a rim. Chaetic sensillum subtype I has a shaft flattened in cross-section, lightly marked with longitudinal grooves and ends with a sharp tip. These sensilla seem to be more numerous than the other subtypes (Figure 5B) and are clearly discernible on the scape and pedicel. Chaetic sensillum subtype II has an apparently smooth shaft surface, a relatively sharp tip and a geniculate shaft, bent just above its insertion on the antennal cuticle (Figure 5C). These sensilla are found on the subdorsal side of the antenna. Chaetic sensillum subtype III has a shaft thicker than subtype I, with a furrowed surface and a rounded tip (Figure 5D).

The basiconic sensilla are peg-shaped with a short shaft (<10 μm long), tapering to a blunt tip (Figure 5D,F). Each has longitudinal grooves and is recessed into its own rimless socket. Basiconic sensilla of subtype III, which were observed on the scape, are short and have a smooth surface (Figure 5C). Basiconic sensilla of subtype II, which arise from an “eyelid”-shaped socket, have a grooved surface and a blunt tip with distal and subdistal depressions resembling pores (Figure 5D, 6A,C). The number of these sensilla increases from article V to X on the ventral and subdistal sides. Males seem to have more basiconic sensilla than do females. The abundant presence of matrices near and at the base of these sensilla suggests that they are involved in the rubbing action of the antennae on the beetle’s surfaces (like that made by the males on the pronotum of the females during mating) and on other surfaces. The abundance of these sensilla on male antennae implies that short-range contact cues can be significantly perceived for mate recognition. Their role in host location and recognition has to be considered, given the fact that females possess basiconic sensilla in the same position even though they have fewer of them.

**Figure 6:**
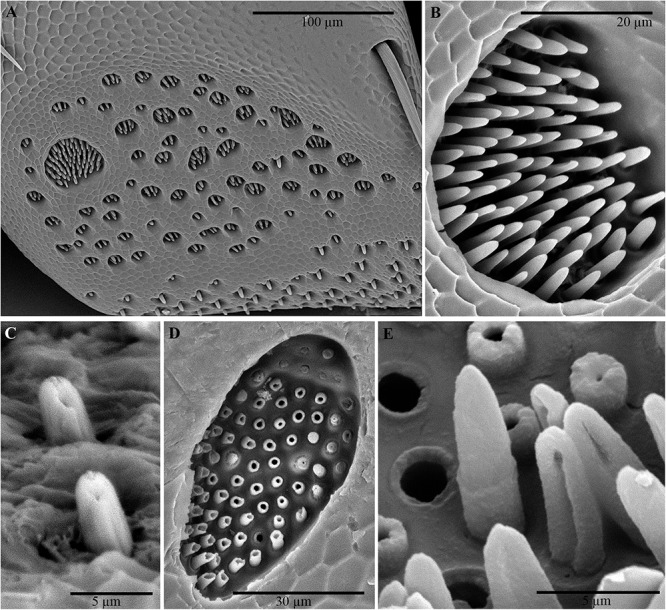
Scanning electron micrographs of antennae of *C. tenebrionis*: antiaxial view of article IX of a female showing several sensorial pits with coeloconic sensilla on the upper side and basiconic sensilla subtype II on the lower side **(A)**; detail of the coeloconic sensilla into a large pit **(B)**; basiconic sensilla subtype II on the antiaxial side of the article VII of a male **(C)**; cross dissected coeloconic sensilla in a sensorial pit on the paraxial side of the article IX of a female **(D)**; detail of dissected coeloconic sensilla on the antiaxial side of the article XI of a male **(E)**.

The coeloconic sensilla – short, smooth-sided pegs with no distinct sockets at their base – are found in recessed cuticular pits located on the distal surface of articles (Figure 6A,B). The distribution of coeloconic sensilla is similar in both sexes. The number of pits on an antenna and for the same gender varies. Each sensorial pit can contain from 1 to 98 short sensilla. The number of these pits increases from articles VI to X and decreases on XI. On the paraxial side, the pits are more numerous in females than in males. A few pits (1–3) are also visible on the distal side (i.e., the side of an article opposing the side of the more distal subsequent article) of each articles IV–XI and increase their size until they reach article X. The porous walls of coeloconic sensilla (Figure 6D,E) suggest an olfactory chemoreceptive role.

### SEM Examination of the Pronotal Resin Copy

**Figure 7:**
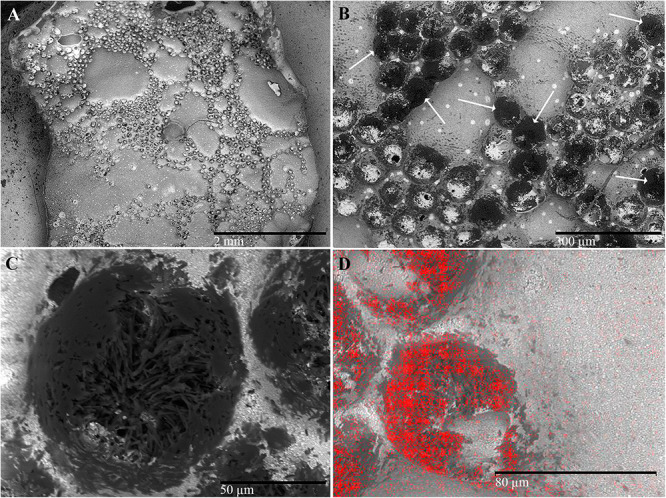
Scanning electron micrographs of *C. tenebrionis*: general view of the resin copy **(A)**, detail of a group of glandular openings **(B)**, and a single opening **(C)** in which bundles of filaments are clearly visible; **(D)** evidence from an energy-dispersive spectrometer showing that the resin surface of a circular area corresponds to the gland opening (carbon detection is red in color). Circular areas (shown by arrows), more or less electron dense, refer to the porous surface of the pronotum.

The resin applied on the pronotum of each beetle produced a “negative” copy of the pronotum surface (Figure 1, 7A) and removed part of the white powder secreted on that surface (Figure 7B,D). On this copy, the rough fields assumed the shape of circular craters with electron-dense borders (Figure 7B,C). They delimited groups of electron-dense filaments (Figure 7B,C) containing a high percentage of carbon (Figure 7D). A sparse, random distribution of small electron-lucent spots was also observed all around the copy on both rough and smooth fields (Figure 7B). These spots may correspond to the simple pores sparsely distributed on the outer surface of the pronotum. According to the energy-dispersive spectra, the highest percentage of carbon (C) was detected on the electron-dense areas of the copy corresponding to the rough fields (82.3% vs. 39.5%), while the percentage of all other elements [barium (Ba), aluminum (Al), silicon (Si), carbon and oxygen (O), constitutive compounds of the resin] was the highest on the areas corresponding to the smooth fields of the pronotum (figure not shown).

### Pre-mating and Mating Behavior

**Figure 8:**
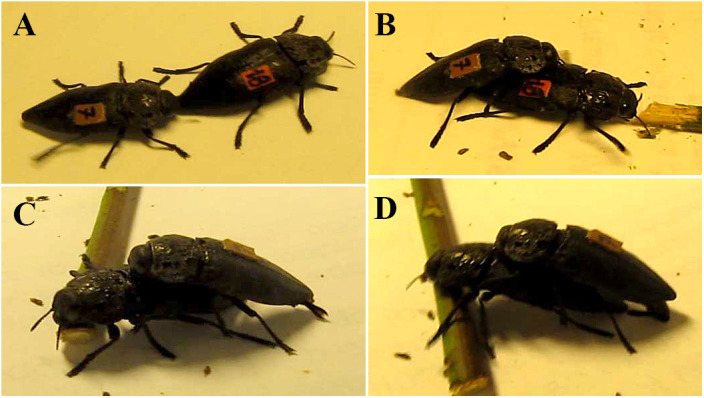
Mating behavioral sequence of *C. tenebrionis*: male oriented toward the female, stopped walking and aligned his body with the female’s – steps 1, 2, and 3 **(A)**, male mounted female – step 4 **(B)**, male touched female pronotum and extruded the aedeagus and started the coupling – steps 5 and 6 **(C,D)**.

Males adopted the following behavioral sequence (Figure 8A–D) when successfully mated with females whose pronota were not coated with resin:

(1) The male oriented himself toward (turned toward) the female, usually moving via the shortest path toward her, but never proceeding on the line she had previously followed;
(2) The male stopped walking (its movement was arrested). At this time, the male became motionless unless the female began to move, in which case, the male suddenly sped up and quickly reached the female;
(3) The male aligned his body with the female’s;
(4) The male mounted the female from her posterior end, touching the cuticle of her dorsal side with his antennae as he moved up her body;
(5) Once the male had reached the pronotum, with his antennae, he extruded the aedeagus and moved his antennae rapidly from side to side across the female’s pronotum;
(6) He inserted the aedeagus into the female’s genital opening. The male rested his antennae on the pronotum of the female and on the first and second pair of legs on the elytrae, while the third pair of legs was usually placed on the substrate. For most of the time, both individuals remained motionless during copulation. If the female attempted to move, the male started shaking his body and moving his antennae quickly;
(7) If the female began to wiggle, the male quickly beat her pronotum with his antennae. When mating ended, the female started to move away while the male dismounted from her dorsum.

Among all the assayed coupling combinations, 76.0% of the assayed females (19 out of 25) and 86.4% of the assayed males (19 out of 22) were able to mate successfully at least once, and four males mated twice during the brief experimental period. All mated males displayed the above-described sequence. Unmated males performed steps 1 to 5, stopping their pre-mating activity when they touched the females’ pronota. Additionally, mating was assayed from 10 a.m. till 5 p.m. on subsequent days within 2 weeks; no difference was observed in the behavior and success of the individuals during that period of time.

Copulation lasted from 0:52 to 13:26 min, averaging about 5:28 ± 3:05 (SD) min. This duration seemed to be unaffected by the size of the individuals. There was no correlation among mating duration, male and female body size (rduration/male = -0.03; rduration/female = 0.36; rmale/female = 0.22, *n* = 19, *p* < 0.05).

Females with half of their pronota coated by resin were still attractive and all assayed males performed steps 1 to 6 (the experiment was stopped at step 6). This assay confirmed that the resin did not interfere with mate receptivity and validated the procedure. Males performed steps 1 to 4 when paired with a female whose pronotum was entirely coated by resin. In this case, instead of proceeding to step 5, males left the females. When they returned, they performed again only steps 1 to 4. In this condition, males were not able to copulate with females. Finally, after the resin was removed, females paired with the same males as they had previously and seemed to be still sexually attractive as determined by the performance of all steps (1–7).

### Chemical Analysis of Pronotum Secretions and Whole Beetle Bodies

**Figure 9:**
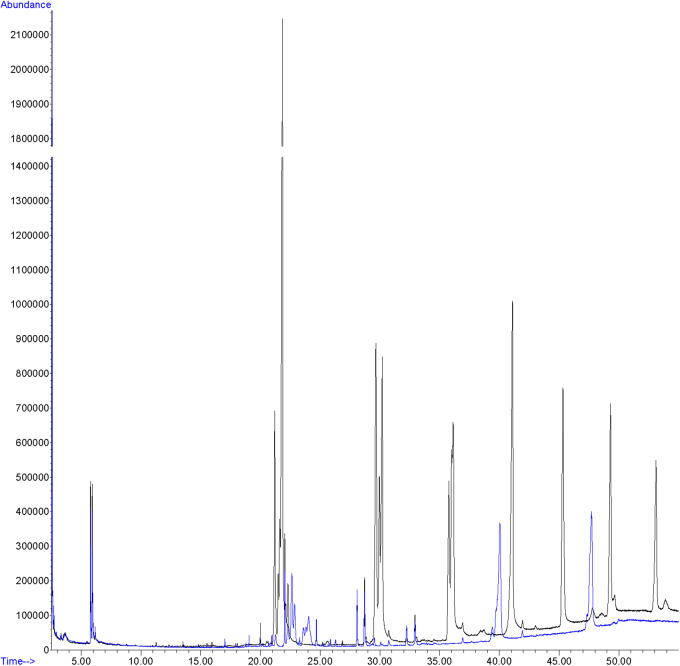
GC profile of cuticular extracts from the whole bodies of virgin females (black line) and virgin males (blue line) of *C. tenebrionis.*

Hexane extracts from resin copies of the female and male pronota contained saturated, unsaturated and branched hydrocarbons in chain lengths ranging from C_8_ to C_36_. In the 2014 assay of the pronotum resin in copies of sexually mature specimens, the chromatograms showed very few qualitative differences between the genders: C_13_ and C_25_ (Table 1) were extracted only from females. With regard to the 2015 assay, GC/MS analysis of the cuticular pronotum extracts identified more peaks in attractive females and attracted males than in virgin and mated adults. In particular, methyl-nonacosane (MeC_29_) and tetratriacontane (C_34_) (Table 1) were not discovered in virgin and mated males. Interestingly, these hydrocarbons were found only in the female cuticular profile. More consistent differences were found in the hydrocarbon profiles of extracts from the whole bodies of male and female virgin adults. The highest peaks for females corresponded to C_23_ (peak 3), C_27_ (peak 22), MeC_29_ (peak 34), and C_34_ (peak 44) (Figure 9 and Table 2).

**Table 1:** Peaks produced by the hexane extracts of resin copies obtained by the pronota of sexually mature male and female pronota in 2014, and by the pronota of attracted males and attractive females in 2015.

	♀	♂	♀	♂
	**Sexually mature**	**Sexually mature**	**Attractive**	**Attracted**

C_8_	+	+	-	-
C_10_	+	+	-	-
C_13_	+	-	-	-
C_14_	-	-	+	-
C_15_	-	-	+	-
Cyclohexene	-	-	+	+
C_22_	-	-	+	-
C_24_	+	+	+	-
C_25_	+	-	-	-
C_26_	+	+	-	+
C_28_	+	+	-	-
MeC_24_	-	-	-	+
MeC_25_	-	-	-	+
C_30_	+	+	-	-
C_31_	+	+	-	-
MeC_29_	-	-	+	-
C_32_	-	-	-	+
C_34_	-	-	+	-
C_35_	-	-	-	+
C_36_	-	-	+	+

*+ and - indicate, respectively, presence or absence of compounds*.

**Table 2:** Peaks produced by the hexane extracts from the whole bodies of virgin males and females of *C. tenebrionis*.

Peak	Retention	Compound	Wholebody	Wholebody
number	time		♂	♀
1	20.07	C_21_	+	-
2	21.00	C_22_	+	-
3	22.04	C_23_	+	-
4	22.93	3 MeC_23_	+	-
5	23.15	5,9-dimethyl C_23_	+	-
6	23.29	C_24_	-	-
7	23.39	3,7+3,9 dimethyl C_23_	-	-
8	23.80	6MeC_24_	-	-
9	24.05	4MeC_24_	+	-
10	24.43	2MeC_34_	-	-
11	24.68	C_25_	+	+
12	25.19	7MeC_25_	-	-
13	25.53	5MeC_25_	+	-
14	25.84	3MeC_25_	+	+
15	26.00	5,9 dimethyl C_25_	+	-
16	26.29	C_26_	+	+
17	26.42	3,7+3,9 MeC_26_	+	-
18	26.85	10+12 MeC_26_	+	-
19	27.10	6 MeC_26_	+	-
20	27.31	4 MeC_26_	+	-
21	27.58	2 MeC_26_	+	-
22	28.11	C_27_	+	+
23	28.71	11+13 MeC_27_	+	-
24	28.93	7 MeC_27_	+	-
25	29.11	5 MeC_27_	+	-
26	29.40	11,13 MeC_27_	+	-
27	29.53	3 MeC_27_	+	+
28	30.07	C_28_	+	+
29	30.19	3,7 dimethyl C_27_	+	-
30	30.68	12+14 7 MeC_28_	+	-
31	31.06	6 MeC_28_	+	-
32	31.27	4 MeC_28_	+	-
33	31.70	2 MeC_28_	+	-
34	32.29	C_29_	+	+
35	32.93	7 MeC_29_	+	+
36	33.57	11,15 6 MeC_29_	+	-
37	33.92	3 MeC_29_	+	+
38	34.59	C_30_	+	-
39	34.71	3,11 dimethyl C_30_	+	-
40	35.74	C_31-ene_	+	-
41	36.96	C_31_	+	+
42	37.66	13 MeC_31_	+	+
43	41.94	C_33_	+	+
44	45.08	C_34_	+	-
45	47.70	11,15 dimethyl C_35_	+	+
46	48.50	MeC_35_	+	+
47	49.65	12+14+16 MeC_36_	+	-
48	53.10	12,22 dimethyl C_36_	+	+

*+/- indicates the presence or absence of the relative compound in extracts of male and female bodies*.

### Constitutive Volatile Profile of *Prunus armeniaca* Leaves

The applied dynamic headspace sampling, combined with TD-GC-MS analysis, allowed to directly collect and identify a total of 33 volatiles from the emission of intact leaves of *P. armeniaca* (Table 3). The detected profile includes a wide range of acids, alcohols, aldehydes, esters, ketones, and terpenes. Although many fruit trees of the Rosaceae family are considered low terpene emitters (Rapparini et al., 2001; Staudt et al., 2010), in the leaf emission profile of *P. armeniaca*, we identified several compounds which belong to this chemical class: C_10_-monoterpene (E)-β-ocimene, C_16_-homoterpene (*E*)-4,8-dimethyl-1,3,7-nonatriene (DMNT) and C_15_-sesquiterpene (*E,E*)-α-farnesene. Traces of other monoterpenes, i.e., β-myrcene and linalool, were also detected. Another class of volatiles emitted by apricot leaves comprises benzenoids (e.g., benzoic acid, benzyl alcohol, benzaldehyde, benzothiazole), which are derived from the aromatic amino acid phenyl alanine. The identified alkanes and organic acids are typical constituents of leaf epicuticular waxes. The profile of emitted volatiles from apricot leaves shows the emission of the phenylpropene methyl eugenol and of the terpenoid cleavage product geranyl acetone.

**Table 3:** Volatile compounds identified from leaves of *P. armeniaca* based on TD-GC-MS measurements.

Compounds	RT	Formula
***Acids***		
Acetic acid	9.09	C_ 2_H_4_O_2_
Hexanoic acid	29.45	C_6_H_14_O_2_
Octanoic acid	41.46	C_8_H_16_O_2_
Nonanoic acid	46.54	C_9_H_18_O_2_
Decanoic acid	50.11	C_10_H_20_O_2_
***Alcohols***		
1-Butanol	10.16	C_ 4_H_10_O
2-Butoxy ethanol	23.41	C_ 6_H_14_O_2_
2-Ethyl 1-hexanol	31.81	C_8_H_18_O
***Alkanes***		
Decane	30.36	C_ 10_H_22_
Undecane	37.08	C_11_H_24_
***Aliphatic aldehydes***		
Octanal	29.32	C_8_H_16_O
Nonanal	36.22	C_9_H_18_O
Decanal	42.24	C_10_H_20_O
***Ketones***		
Cyclohexanone	21.38	C_ 6_H_10_O
2-Heptanone	21.77	C_ 7_H_14_O
6-Methyl-5-hepten-2-one	23.20	C_8_H_14_O
***Esters***		
3-Ethoxy ethyl propanoate	28.45	C_ 7_H_14_O_3_
(*Z*)-3-Hexenyl acetate	29.93	C_8_H_14_O_2_
2-Butoxy ethyl acetate	35.33	C_8_H_16_O_3_
***Benzenoids***		
Benzaldehyde	25.75	C_ 7_H_6_O
Benzyl alcohol	31.26	C_7_H_8_O
Methyl salicylate	41.47	C_8_H_8_O_3_
Benzothiazole	42.71	C_7_H_5_NS
***Terpenes***		
β-Myrcene	26.40	C_10_H_16_
(*E*)-β-Ocimene	33.05	C_10_H_16_
Linalool	36.33	C_10_H_18_O
(*E*)-4,8-Dimethyl-1,3,7-nonatriene (DMNT)	37.90	C_11_H_18_
(*E,E*)-α-farnesene	54.70	C_15_H_24_
***Others***		
Cyclohexane isothiocyanate	42.38	C_ 7_H_11_NS
Methyl eugenol	50.68	C_11_H_14_O_2_
Geranyl acetone	52.60	C_11_H_18_O_2_

### Olfactometer Assays With Plant VOCs

Responses were usually distinct between adults according to gender. The females were attracted most by (Z)-3-hexen-1-ol and 3-methyl-butanol, whereas the males were attracted most by 3-methyl-butanol and 1-pentanol (Table 4). Benzaldehyde and 2-hexanone for the females, and s-limonene, (Z)-3-hexen-1-ol and 2-hexanone for the males appeared to act as repellents (Table 4).

**Table 4:** Response index of females (F, *n* = 15) and males (M, *n* = 15) of *C. tenebrionis* to some VOCs.

VOCs	*C. tenebrionis*	
	Male	Female
(Z)-3-Hexen-1-ol	-13.33	46.67
1-Pentanol	6.67	-13.33
Benzaldehyde	0	-33.33
3-Methyl-butanol	26.67	26.67
β-Myrcene	-6.67	-6.67
2-Hexanone	-13.33	-26.67
s-Limonene	-40.00	-13.33

### Identification of Candidate Chemosensory Genes

Sequencing and *de novo* assembly of the transcriptomes derived from the antennae and whole bodies of *C. tenebrionis* adult males and females led to the identification of 51,394 nucleotide sequences (contigs). These contigs, each of which encodes for a putative protein characterized by a specific function, were functionally annotated using the Blast2GO software^3^. Our analysis allowed the identification of 14 putative OBPs and 11 putative CSPs (Table 5). The alignment of the 14 identified *C. tenebrionis* OBPs and the 11 identified *C. tenebrionis* CSPs is shown in Supplementary Material S1. Furthermore, analysis of the identified putative OBP and CSP genes using the RPKM method, allowed for the evaluation of expression level differences between adult males and females (measured in antennae and whole bodies) (Figure 10).

**Table 5:** List of the identified odorant-binding proteins (OBPs) in *C. tenebrionis* adult males and females and list of the identified chemosensory proteins (CSPs) in *C. tenebrionis* adult males and females.

Unigene reference	Gene name	ORF (bp)	BLASTx annotation	*E*-value	AA identity (%)
Capnodis_C409	*CtenOBP1*	411	Odorant-binding protein 10 (*Agrilus mali*)	3e-47	59
Capnodis_C789	*CtenOBP2*	681	PREDICTED: general odorant-binding protein 67-like (*Aedes albopictus*)	5e-07	28
Capnodis_C4812	*CtenOBP3*	450	General odorant-binding protein 19d (*Agrilus planipennis*)	1e-41	54
Capnodis_C7413	*CtenOBP4*	405	Uncharacterized protein LOC108739404 (*Agrilus planipennis*)	2e-49	68
Capnodis_C8196	*CtenOBP5*	393	General odorant-binding protein 83a isoform X2 (*Agrilus planipennis*)	4e-55	65
Capnodis_C8312	*CtenOBP6*	531	PREDICTED: general odorant-binding protein 70 isoform X1 (*Tribolium castaneum*)	8e-38	53
Capnodis_C12606	*CtenOBP7*	423	Odorant-binding protein 3 (*Agrilus mali*)	2e-45	60
Capnodis_C16094	*CtenOBP8*	453	General odorant-binding protein 19d (*Agrilus planipennis*)	2e-31	45
Capnodis_C17506	*CtenOBP9*	408	Odorant-binding protein (*Dendrolimus kikuchii*)	8e-11	35
Capnodis_C17816	*CtenOBP10*	642	OBP16 (*Holotrichia parallela*)	7e-11	26
Capnodis_C18027	*CtenOBP11*	411	PREDICTED: general odorant-binding protein 56d-like (*Habropoda laboriosa*)	7e-18	34
Capnodis_C21011	*CtenOBP12*	831	General odorant-binding protein 71 (*Agrilus planipennis*)	1e-29	45
Capnodis_C34461	*CtenOBP13*	189	Odorant-binding protein 3 (*Agrilus mali*)	6e-33	54
Capnodis_C43694	*CtenOBP14*	399	Odorant-binding protein 7 (*Agrilus mali*)	4e-12	32
Capnodis_C4	*CtenCSP1*	240	Ejaculatory bulb-specific protein 3 (*Agrilus planipennis*)	1e-39	73
Capnodis_C120	*CtenCSP2*	369	Chemosensory protein 2 (*Agrilus mali*)	4e-37	53
Capnodis_C856	*CtenCSP3*	393	Ejaculatory bulb-specific protein 3 (*Agrilus planipennis*)	5e-49	74
Capnodis_C857	*CtenCSP4*	393	Ejaculatory bulb-specific protein 3 (*Agrilus planipennis*)	9e-50	74
Capnodis_C2805	*CtenCSP5*	399	Chemosensory protein 1 (*Agrilus mali*)	8e-63	83
Capnodis_C6098	*CtenCSP6*	366	Ejaculatory bulb-specific protein 3-like isoform X1 (*Agrilus planipennis*)	8e-56	84
Capnodis_C6962	*CtenCSP7*	429	Ejaculatory bulb-specific protein 3-like (*Agrilus planipennis*)	3e-56	72
Capnodis_C7044	*CtenCSP8*	396	Chemosensory protein 4 (*Agrilus mali*)	2e-66	76
Capnodis_C7625	*CtenCSP9*	1005	Chemosensory protein 7, partial (*Agrilus mali*)	4e-68	81
Capnodis_C12217	*CtenCSP10*	330	Chemosensory protein 8 (*Agrilus mali*)	1e-53	92
Capnodis_C30511	*CtenCSP11*	303	Uncharacterized protein LOC108734435 (*Agrilus planipennis*)	5e-43	69

**Figure 10:**

Heat map showing differences in the expression of OBPs between *C. tenebrionis* adult males and females (measured in antennae and whole bodies) **(A)** and heat map showing differences in the expression of CSPs between *C. tenebrionis* adult males and females (measured in antennae and whole bodies) **(B)**. The housekeeping genes RPS18 and EF1-alpha are used for normalization and have been shown to confirm the uniform expression of these control genes across samples. The map is based on log_2_-transformed RPKM values shown in the gradient heat map (blue represents weakly expressed genes, and red represents strongly expressed genes).

Our results showed that 13 out of the 14 identified putative OBPs and all the 11 identified putative CSPs displayed highest expression levels in the antennae of *C. tenebrionis* males and females.

## Discussion

The examination of *C. tenebrionis* using light and SEMicroscopy revealed pores on the inner and outer surfaces of the pronotum of both genders. The pore arrangement was not perfectly symmetrical and porous fields were larger in males than in females when the surfaces of whole pronotum were compared. No further differences were found between the genders. Whether other jewel beetles share the unexpectedly large porous fields found on the pronota of male *C. tenebrionis* is unknown. Gland pores were previously detected on pronota of other male Coleoptera, including many long-horned beetles, and in some species were related to sex or aggregation pheromone secretions (Noldt et al., 1995; Ray et al., 2006; Hanks et al., 2007; Lacey et al., 2007; Hanks and Millar, 2016). Behavioral assays on *C. tenebrionis* did not point to the secretion of aggregation cues by males or females (Bari et al., 2004; Sharon et al., 2010), which is confirmed by field observations (Bonsignore and Jones, 2013). On the contrary, pre-mating and mating behavior assigns a relevant role to females’ pronota of this species. Males were unable to recognize the sexual suitability of females with pronota that had been entirely coated even though they did find and mount the females. These data suggest that males need confirmation of the readiness of a female to mate. Cuticular hydrocarbons or other cues have to be secreted on the pronota of sexually mature females of *C. tenebrionis*. These cues can be perceived by the contact chemosensilla of the male antennae during the mate recognition process as was shown for *A. planipennis*, although the location of secretory gland openings are currently unknown (Silk et al., 2009; Silk and Ryall, 2015). Perhaps the volatile cues involved in male attraction and orientation are perceived even when the female’s pronotum is coated. Moreover, males may exploit other means (e.g., in our experimental model, visual) to find females and plant-emitted compounds may also be involved (Sharon et al., 2010; Bonsignore and Jones, 2013) as has been observed for *A. planipennis* (Silk and Ryall, 2015).

The SEM study identified chaetic, basiconic and coeloconic sensilla on the antennae. The morphology of *C. tenebrionis* antennae is in agreement with Volkovitsh (2001). Sensorial pits containing coeloconic sensilla increase in number according to the article’s distance from the antennal base. Basiconic sensilla placed on the ventral margin of the antennae possess the morphological characteristics of contact chemosensilla that could be involved in testing substances on touched surfaces. Sensorial pits (also referred to as “fossae” by Volkovitsh, 2001) containing fields of coeloconic sensilla are common among buprestid species and have been used to develop the systematics of the taxon (Volkovitsh, 2001). In the numerous examples shown by Volkovitsh (2001), uniporous sensilla and several types of basiconic sensilla dominate.

The pre-copulatory behavior observed in the current study and adopted by males for finding females in a small mating arena, and the potential presence of a pheromone (Bari et al., 2004) may imply the involvement of a short-distance cue. This hypothesis is supported by studies that suggest males of *A. planipennis* were able to find females at short distance (≤5 cm) through a short-range volatile pheromone (Pureswaran and Poland, 2009; Silk and Ryall, 2015). It is worth noting that *C. tenebrionis* adults tend to aggregate especially when exposed directly to sunlight (Bonsignore et al., 2008; Bonsignore and Jones, 2013). This exposure and the black color of the body can increase body temperature (Garzone et al., unpublished observations) and stimulate the evaporation of less volatile chemicals secreted by the beetle. Both factors might contribute to the aggregation tendency, and the way mates are found and sexual maturity perceived at a short distance.

The biologically inert resin was used for the current bioassays and did not cause any repellence or interference with the beetle behavior. The fluid resin was applied to a body part (pronotum, in our case) and, after hardening, easily removed. It detached compounds from the surfaces of the insects without harming or killing them. Compounds detached by the resin were organic compounds, according to the EDS analysis, and likely came from glandular secretions and the outermost cuticular layers. Previous analytical studies have been performed on solid-phase microextraction fibers (SPMEs) and solvent extraction of parts or whole body of beetles belonging to Buprestidae and few other taxa (Fukaya, 2003; Ginzel et al., 2006; Lelito et al., 2009; Silk et al., 2009; González-Núñez et al., 2012; Yew and Chung, 2015; Yang et al., 2017; etc.). Hydrocarbons are the major components of the cuticular coating of adult insects (Lockey, 1991), even though a wide range of more polar lipids have also been found and shown to have attractive properties (Yew and Chung, 2015; Hanks and Millar, 2016). The cuticular organic compounds sampled from the pronotum resin copies of attracted males and attractive females, and among the extracts of the whole bodies of virgin males and females of *C. tenebrionis*, appeared to differ qualitatively. These differences could support the assumption that the pronota of attractive females can emit cues that play specific roles in pre-mating and require further investigations in comparing sexually immature and mature females. These differences partially support the results of Cui et al. (2018); they found many more expressed OBPs in male antennae of *Agrilus mali* Matsumura than in female ones and these OBPs showed strong binding affinity to C_2-15_ compounds. In the current study, C_13_ and C_14-15_ were found only in the solvent extracts of the pronotum resin copies of sexually mature females and of attractive females, respectively. Current data are not in accordance with González-Núñez et al. (2012), who did not discover qualitative differences between males and females of *C. tenebrionis* in the levels of more than 40 cuticular hydrocarbons extracted by solvent from the whole body of both genders. The cuticular extracts did not elicit any male behavior at the time of mating, leading to the assumption that there is no contact pheromone in *C. tenebrionis*. Our finding – that methyl-nonacosane and tetratriacontane peaks only in the pronotum resin copies of females attracting males of *C. tenebrionis* – is in agreement with that of Lelito et al. (2009). They found 3-methyl-tricosane in limited quantities on the cuticle of young females of *A. planipennis*, and the levels of this chemical increased with the beetle’s age and achievement of sexual maturity. Mfarrej and Sharaf (2011) recognized three aliphatic hydrocarbons produced by adults of *Capnodis carbonaria* (Klug.): hexacosane was found in the male cuticle, *n*-heptacosane in the female body and nonacosane in the male hindgut. The female body extract *n*-heptacosane was the most active hydrocarbon used to trap *C. carbonaria* adults. In the current research, hexacosane, heptacosane, and nonacosane were extracted from the bodies of both genders (peaks 16, 22, 34, Table 2) and their biological activity needs to be investigated. Finally, 9-methyl-pentacosane was found to be the main component of the contact pheromone in *A. planipennis* (Silk and Ryall, 2015).

Unlike males, females of *C. tenebrionis* respond highly positively in a preliminary olfactometer assay to (Z)-3-hexen-1-ol, suggesting the compound is involved in host finding. A high electron-antennographic response was previously shown for this green leaf volatile in *C. tenebrionis* (Bari et al., 2004). The current data on behavioral differences between males and females appear to contradict earlier data showing more virgin males than virgin females in oaks infested by *A. biguttatus* (F.) (Silk and Ryall, 2015; Vuts et al., 2016), but the numbers are in accordance with the data on *Coroebus florentinus* Herbst. (Furstenau et al., 2012) and *A. planipennis* infesting cork oak (Silk and Ryall, 2015).

Analysis of the volatile emission profile of healthy apricot plants increases the pool of chemicals to be assayed under olfactometer and electron-antennography in order to evaluate their influence on the behavior of adult *C. tenebrionis*. Although most of *Prunus* species are considered low VOC emitters (Baraldi et al., 1999; Rapparini et al., 2001; Karlik et al., 2002; Staudt et al., 2010), some of the constitutive volatiles released at leaf level may play a key functional role for long-lived adults of *C. tenebrionis* in host locations. If the host foliage of fruit trees may be an important point of this plant-insect interaction, leaf volatiles are used as olfactory cues by several buprestid species belonging to the *Agrilus* genus to locate feeding and mating sites in natural forests (Koch et al., 2015; Vuts et al., 2016, and references therein). The current data represent a basic profile of healthy plants and requires to be compared with that of plants stressed by biotic and abiotic factors which can favor the emission of more attractive VOCs for the beetle.

Recent findings on the relationship between buprestid beetles and trees showed that the apple buprestid *A. mali* use multiple OBPs to discriminate among compounds belonging to different chemical families, showing high affinity for alcohols, esters and terpenes (Cui et al., 2018). Among terpenes, the observed leaf emissions of (*E*)-β-ocimene, (*E*)-4,8-dimethyl-1,3,7-nonatriene (DMNT) and (E,E)-α-farnesene from apricot leaves may serve as relevant signals for the buprestid *Capnodis.* These compounds have been found to be the principal volatiles in the constitutive emissions from leaf-bearing shoots of different *Prunus* genotypes (Staudt et al., 2010) and other Rosaceae (Reidel et al., 2017; Yang et al., 2018), including fruit species frequently attacked by *C. tenebrionis*. These volatiles are commonly induced by herbivory in different trees, including rosaceous fruit species (Staudt et al., 2010; Giacomuzzi et al., 2016, 2017; Copolovici et al., 2017). The volatiles (*E*)-β-ocimene and DMNT, identified in leaf emissions of *Quercus robur*, showed positive electroantennography (EAG) responses in adult buprestid *A. biguttatus* (Vuts et al., 2016). In another fruit tree-insect relationship, DMNT and the monoterpene linalool have been found to elicit a high EAD response from adult females of a common apple moth (Lepidoptera: Totricidae). These two terpenes are often considered key volatiles in herbivore deterrence (Borrero-Echeverry et al., 2015).

Volatile benzenoids, which are qualitatively abundant in the emission profile of the analyzed, healthy apricot plants, play important roles in plant communication with the environment (Dudareva et al., 2013). Among them, benzaldehyde is considered a breakdown product of prunasin, a cyanogenic glycoside commonly found in *Prunus* species and other Rosaceae (Santamour, 1998). This aromatic aldehyde represents a key compound in plant–insect interactions of several fruit tree species of Rosaceae (Staudt et al., 2010; Najar-Rodriguez et al., 2013; Lu et al., 2015), but its attraction was not confirmed in the current olfactometer assay.

Interestingly, the release of methyl eugenol and geranyl acetone from intact apricot foliage was never previously detected in the leaf emission of other *Prunus* species. Geranyl acetone is a carotenoid-derived compound that in stone-fruit species has been previously detected only in the volatile emissions of fruits and flowers of *P. cerasifera* (Reidel et al., 2017). Unique compounds may contribute to the chemical fingerprint of plant hosts and may play a key role in insect recognition of plant odors.

The identification of the C_6_-ester (*Z*)-hexenyl acetate and nonanal from intact, healthy leaves of apricot plants confirms previous findings on leaf emissions from apricot (Yang et al., 2018) and other fruit tree species of Rosaceae (Najar-Rodriguez et al., 2013; Giacomuzzi et al., 2017; Reidel et al., 2017). Although this C_6_-ester is frequently present when the corresponding alcohol (*Z*)-3-hexenol is emitted by plants, as both are products of the same lipoxygenase pathway (Dudareva et al., 2013), we did not detect this C_6_-alcohol in the emissions of intact and healthy plants. Typically, C_5_- and C_6_-alcohols, together with related aldehydes, are produced and emitted after tissue damage (Dudareva et al., 2013). Recent findings on field-grown apricot plants showed induced emissions of (*Z*)-3-hexenol at leaf level in response to Coleoptera beetles (Scolytidae), which typically attack fruit trees of Rosaceae (Yang et al., 2018).

If the majority of the constitutive leaf volatiles identified in apricot is widespread in both pome- and stone-fruit trees of Rosaceae, leaf volatiles that are induced by both abiotic and biotic factors may be also relevant for the ability of *C. tenebrionis* to locate host trees. In a previous study, volatiles emitted from stressed nitrogen-rich peach plants enhanced the attractiveness of *C. tenebrionis* females (Sharon et al., 2010). Intraspecific variability in aphid-induced emissions rates of several VOCs, including (*E*)-β-ocimene, DMNT and (*E,E*)-β-farnesene, were associated with the resistance traits of different *Prunus* genotypes to aphid attack (Staudt et al., 2010). However, in another tree-insect system involving the buprestid *A. planipennis*, the lack of an association between insect resistance and intraspecific differences in leaf VOCs emission was shown (Koch et al., 2015). Whether the VOCs identified in our investigation confer direct or indirect protection to apricot trees against *C. tenebrionis* remains to be established.

The exact function of each olfactory gene family in Coleoptera is still not completely known, as few studies have yet been performed on the genes involved in olfaction in coleopterans (Engsontia et al., 2008; Mitchell et al., 2012; Andersson et al., 2013; Antony et al., 2016). Because many OBPs, CSPs and ORs isolated and characterized in moths, dipterans and hemipterans have been shown to have affinities to host plant volatile compounds or pheromones (He et al., 2011; Harada et al., 2012; Yin et al., 2012), the exact function of these proteins in coleopteran communication is starting to be studied. Mamidala et al. (2013) performed a complete studies on candidate genes involved in the perception, processing and degradation of volatiles in the invasive buprestid *A. planipennis*, identifying 9 OBPs, 2 ORs, 1 SNMP, 6 IRs, 6 ionotropic glutamate receptors (IGluRs), 2 GRs and 4 CSPs in the antennal transcriptome. The identified number of odorant genes provided insight into the olfactory processes of *A. planipennis* in detecting host- and mate-locating cues. Cui et al. (2018) cloned and characterized by binding assays two OBPs, both of which were identified in the antennal transcriptome of the apple buprestid beetle *A. mali*. In our study, 14 OBPs and 11 CSPs were identified in the antennae, the main organs involved in chemoreception in which soluble olfactory proteins are commonly highly expressed, and body transcriptome of adult males and females of the invasive buprestid *C. tenebrionis*. All the identified CtenOBPs were shown to share over 26% residues with OBPs from other coleopterans, although insect OBPs commonly share only 10–15% of their residues between species (Pelosi et al., 2017). The BLASTx results indicated that 4 CtenOBPs showed amino acid identities (32–60%) with *A. mali* OBPs. In particular, CtenOBP7 and CtenOBP13 shared 60% and 54% amino acid identity. respectively, with OBP3 of *A. mali* (AmalOBP3). AmalOBP3 was functionally characterized in competitive fluorescence-binding assays (Cui et al., 2018) and showed binding affinity toward both (Z)-3-hexenol and 3-methyl-1-butanol. In our work, (Z)-3-hexenol and 3-methyl-1-butanol attracted females and both adult genders of *C. tenebrionis*, respectively, in olfactometer assays. In addition, both CtenOBP7 (Capnodis_C12606) and CtenOBP13 (Capnodis_C34461) were most highly expressed in antennae (Figure 10). The high amino acid sequence similarity between CtenOBP7, CtenOBP13 and AmalOBP3, as well as the binding affinity of AmalOBP3 with two molecules that we showed to be attractive for the insect and the high expression level of CtenOBP7 and CtenOBP13 provide the basis for our hypothesis that in *C. tenebrionis* these OBPs may be involved in the recognition of (Z)-3-hexenol and 3-methyl-1-butanol. Furthermore, the higher expression of CtenOBP7 and CtenOBP13 in antennae than in the whole body suggests that these OBPs may play important roles in the detection of general odorants such as the host plant volatiles as reported in other insect species (Landolt, 1997; Smyth and Hoffmann, 2003). Moreover, 5 of the 14 identified CtenOBPs shared high amino acid identities (45–68%) with *A. planipennis* OBPs at NCBI. Gas chromatography-electroantennogram detection bioassays demonstrated that *A. planipennis* antennae were highly responsive to (Z)-3-hexenol (de Groot et al., 2008). The high amino acid sequence similarity between the 5 CtenOBPs and *A. planipennis* OBPs suggests that these CtenOBPs may be involved in the detection of host plant volatiles. In order to determine the exact binding mechanisms between soluble olfactory proteins in *C. tenebrionis* and putative ligands, extensive studies with chemical ligands are needed since multiple OBPs and CSPs are often used in the mechanisms of insects’ chemical perception (Hekmat-Scafe et al., 2002; Zhou, 2010). The biochemical characterization of CtenOBPs and CSPs will contribute to increasing our knowledge of the molecular mechanisms underlying the complex chemical communication system in this insect.

This study on the intraspecific and interspecific interactions in *C. tenebrionis* has summarized some aspects of the morphology of the insect’s pronotum and antennae, on its pre-copulatory behavior and on beetle–plant communication; understanding the chemical ecology of this pest will be useful in the development of environmentally friendly management strategies for its control. The information collected to date seems to pair the chemical ecology of *C. tenebrionis* with that of *A. planipennis* (Silk and Ryall, 2015).

## Ethics Statement

Insects used in this work were treated as well as possible given the constraints of the experimental design.

## Author Contributions

PF and AS contributed to the design and implementation of the research and analysis of the results on the identification of candidate chemosensory genes with support from SB and RS. EdL contributed to the design and implementation of the research, and to the analysis of the results on the morphology of antennae and pronotum, pre-mating and mating behavior, and olfactometer assays with support from GB, VG, PV, and CY. FR designed and performed the experiments on the VOC emission from plants with support from OF and RB. FR performed the TD-GC-MS analyses of VOCs. AA contributed at the GC-MS analysis of beetle extractions and at the interpretation of the results. HV performed the *de novo* transcriptome assembly and analysis. EdL, PF, FR, and AS supervised the research, interpreted the data, and wrote the manuscript with contributions from all other authors.

## Conflict of Interest Statement

CY is currently employed by company Syngenta in Turkey. CY was a masters student at CiHEAM IAM-B while undertaking part of this study which was performed at the University of Bari Aldo Moro. The remaining authors declare that the research was conducted in the absence of any commercial or financial relationships that could be construed as a potential conflict of interest. Syngenta had no role in study design, data collection and analysis, decision to publish, or preparation of the manuscript.

## Footnotes

1 http://mpgc.mpipz.mpg.de/home/

2 www.blast2go.de

3 www.blast2go.com

## Supplementary Material

MATERIAL S1 Alignment of amino acid sequences of candidate OBPs from *Capnodis tenebrionis* **(A)**. Alignment of amino acid sequences of candidate CSPs from *Capnodis tenebrionis* **(B)**.
