# Corrigendum: Chemical Ecology of *Capnodis tenebrionis* (L.) (Coleoptera: Buprestidae): Behavioral and Biochemical Strategies for Intraspecific and Host Interactions

**DOI:** 10.3389/fphys.2020.00668

**Published:** 2020-06-26

**Authors:** Giuseppe Bari, Andrea Scala, Vita Garzone, Rosanna Salvia, Cem Yalcin, Pasqua Vernile, Antonella Maria Aresta, Osvaldo Facini, Rita Baraldi, Sabino A. Bufo, Heiko Vogel, Enrico de Lillo, Francesca Rapparini, Patrizia Falabella

**Affiliations:** ^1^Department of Soil, Plant and Food Sciences, University of Bari Aldo Moro, Bari, Italy; ^2^Department of Science, University of Basilicata, Potenza, Italy; ^3^Syngenta, Izmir, Turkey; ^4^Department of Chemistry, University of Bari Aldo Moro, Bari, Italy; ^5^Department of Biology, Agriculture and Food Sciences, Biometeorology Institute, National Research Council, Bologna, Italy; ^6^Department of Entomology, Max Planck Institute for Chemical Ecology, Jena, Germany

**Keywords:** chemoreception, mating, mediterranean flat-headed root-borer, scanning electron microscopy, soluble olfactory proteins, volatile organic compounds

In the original article, there was a mistake in Table 1 and Table 2 as published. The Table 1 and Table 2 had incorrect column headers due to an inversion of the symbols ♀ and ♂ with the consequent incorrect interpretation of the symbols + and – within the same Tables. The corrected Table 1 and Table 2 appear below.

**Table 1:** Peaks produced by the hexane extracts of resin copies obtained by the pronota of sexually mature male and female pronota in 2014, and by the pronota of attracted males and attractive females in 2015.

	**♀ Sexually mature**	**♂ Sexually mature**	**♀ Attractive**	**♂ Attracted**
C_8_	+	+	–	–
C_10_	+	+	–	–
C_13_	+	–	–	–
C_14_	–	–	+	–
C_15_	–	–	+	–
Cyclohexene	–	–	+	+
C_22_	–	–	+	–
C_24_	+	+	+	–
C_25_	+	–	–	–
C_26_	+	+	–	+
C_28_	+	+	–	–
MeC_24_	–	–	–	+
MeC_25_	–	–	–	+
C_30_	+	+	–	–
C_31_	+	+	–	–
MeC_29_	–	–	+	–
C_32_	–	–	–	+
C_34_	–	–	+	–
C_35_	–	–	–	+
C_36_	–	–	+	+

*+ and – indicate, respectively, presence or absence of compounds*.

**Table 2:** Peaks produced by the hexane extracts from the whole bodies of virgin males and females of *C. tenebrionis*.

**Peak number**	**Retention time**	**Compound**	**Wholebody ♀**	**Wholebody ♂**
1	20.07	C_21_	+	–
2	21.00	C_22_	+	–
3	22.04	C_23_	+	–
4	22.93	3 MeC_23_	+	–
5	23.15	5,9-dimethyl C_23_	+	–
6	23.29	C_24_	–	–
7	23.39	3,7+3,9 dimethyl C_23_	–	–
8	23.80	6MeC_24_	–	–
9	24.05	4MeC_24_	+	–
10	24.43	2MeC_34_	–	–
11	24.68	C_25_	+	+
12	25.19	7MeC_25_	–	–
13	25.53	5MeC_25_	+	–
14	25.84	3MeC_25_	+	+
15	26.00	5,9 dimethyl C_25_	+	–
16	26.29	C_26_	+	+
17	26.42	3,7+3,9 MeC_26_	+	–
18	26.85	10+12 MeC_26_	+	–
19	27.10	6 MeC_26_	+	–
20	27.31	4 MeC_26_	+	–
21	27.58	2 MeC_26_	+	–
22	28.11	C_27_	+	+
23	28.71	11+13 MeC_27_	+	–
24	28.93	7 MeC_27_	+	–
25	29.11	5 MeC_27_	+	–
26	29.40	11,13 MeC_27_	+	–
27	29.53	3 MeC_27_	+	+
28	30.07	C_28_	+	+
29	30.19	3,7 dimethyl C_27_	+	–
30	30.68	12+14 7 MeC_28_	+	–
31	31.06	6 MeC_28_	+	–
32	31.27	4 MeC_28_	+	–
33	31.70	2 MeC_28_	+	–
34	32.29	C_29_	+	+
35	32.93	7 MeC_29_	+	+
36	33.57	11,15 6 MeC_29_	+	–
37	33.92	3 MeC_29_	+	+
38	34.59	C_30_	+	–
39	34.71	3,11 dimethyl C_30_	+	–
40	35.74	C_31−ene_	+	–
41	36.96	C_31_	+	+
42	37.66	13 MeC_31_	+	+
43	41.94	C_33_	+	+
44	45.08	C_34_	+	–
45	47.70	11, 15 dimethyl C_35_	+	+
46	48.50	MeC_35_	+	+
47	49.65	12+14+16 MeC_36_	+	–
48	53.10	12,22 dimethyl C_36_	+	+

*+/– indicates the presence or absence of the relative compound in extracts of male and female bodies*.

The authors apologize for this error and state that this does not change the scientific conclusions of the article in any way. The original article has been updated.

