# Supplementary material for: Chemical Ecology of Capnodis tenebrionis (L.) (Coleoptera: Buprestidae): Behavioral and Biochemical Strategies for Intraspecific and Host Interactions
Source: Front Physiol. 2019 May 27;10:604. doi: 10.3389/fphys.2019.00604 (PMC6545930; doi:10.3389/fphys.2019.00604)
Supplement: MATERIAL S1 — Alignment of amino acid sequences of candidate OBPs from Capnodis tenebrionis (A). Alignment of amino acid sequences of candidate CSPs from Capnodis tenebrionis (B). [file Table_1.DOCX]

**A**

CtenOBP2 MRASQLFILLTAVVGFSTPYLLPFHETELLPSILTRHR-REAES---------------- 43

CtenOBP10 MGASQVFILLTAIVGFTTSYLLTYDDDQLFPIITLRER-REVHK---------------- 43

CtenOBP6 MA--MKYFLF--------LFVCAVCIEDTFCGILNSINRQNSN----------------- 33

CtenOBP12 MYLITVSFLI--------AFLPAFRTQALECGIGKREHQREVQRIMQICLYNTVGNGNQN 52

CtenOBP3 MKSLAVFFLV--------A----------------------------------------- 11

CtenOBP8 MKSVAIICLS--------F----------------------------------------- 11

CtenOBP5 MKHF---LVI--------F----------------------------------------- 8

CtenOBP7 MKAL---VVI--------F----------------------------------------- 8

CtenOBP13 ------------------------------------------------------------ 0

CtenOBP9 --MLIESMLI--------V----------------------------------------- 9

CtenOBP11 MNRFAVPVLM--------F----------------------------------------- 11

CtenOBP14 MNLYWIAI---------------------------------------------------- 8

CtenOBP1 MKIL-CSVLA--------F----------------------------------------- 10

CtenOBP4 MKSINLLPVL--------L----------------------------------------- 11

CtenOBP2 -----------------------------------------------------------S 44

CtenOBP10 ------------------------------------------------------------ 43

CtenOBP6 -------------------DS----------------------Q----TLSETR--CKIP 46

CtenOBP12 VGHSASAGRDYEVGDSDSSDSSESESAEADSGVQSNDTNRNAYMRLSSTIAPENGNT--S 110

CtenOBP3 -------------------------------------------V-----AVCAG-----L 18

CtenOBP8 -------------------------------------------L----AVVSIK-----A 19

CtenOBP5 -------------------------------------------A----LLPAVL------ 15

CtenOBP7 -------------------------------------------L----LLHLLQGSHSAA 21

CtenOBP13 ------------------------------------------------------------ 0

CtenOBP9 -------------------------------------------V----AFTTVN-----A 17

CtenOBP11 -------------------------------------------I----VLSLVQ-----A 19

CtenOBP14 -------------------------------------------L----TLGSLI-----S 16

CtenOBP1 -------------------------------------------F----LIATVQ-----A 18

CtenOBP4 -------------------------------------------L----VVASVK-----A 19

CtenOBP2 SGLPN-------------HKYEEFSHPHFSCCGEEIVSHLSRNEFYAYINCSREVFAG-- 89

CtenOBP10 ----------------------FLNHSDLPCCGTEIINNLRESEMEVLKKCSEKNGSE-- 79

CtenOBP6 KTAPKNIE------------------NAIVECQVEIQVALLTDAIELIDTTKTEKTDA-T 87

CtenOBP12 STTPQNRQSNQDAFNNSTRQLNQQSGSNANNTRQETA-----QNLQSLQNGTNNNNGGNN 165

CtenOBP3 SISPETLGK-----------LAQKGKEVIKTCKTETGAS--DEDLTVFAG-K-------- 56

CtenOBP8 EIPPETMGK-----------IIQTTQTIIKKCREDTNAN--EDDLSVLSEMK-------- 58

CtenOBP5 CLSEE---------------LQELANMLHKTCQEQTGAA--EEMITNAGKGD-------- 50

CtenOBP7 MSEAQ---------------LQNAVNLMKNVCQPKVKVS--DKLINKMHNGE-------- 56

CtenOBP13 ------------------------------------------------------------ 0

CtenOBP9 ELSPK---------------IKKEFLQHSKECMDETKIS--SQELEDLKNGN-------- 52

CtenOBP11 DLTDE---------------QRQKLKSHHEACSAEFPIS--KENLQALREGK-------- 54

CtenOBP14 ANKEE---------------HSQFLKGIRGICMNEVPIQ--LESLDDFREGR-------- 51

CtenOBP1 DLSDE---------------QKAKLKKHQEECIKLFPVD--KQLLEKARKGD-------- 53

CtenOBP4 ELPPE---------------LQKIVKERQDECLASNPVP--AEILENARQGK-------- 54

CtenOBP2 -RPQGNPSS-----QNKTQRYICFQECFAKKIDLLDNHDVPNRDKIKEHLTRLLK--NTT 141

CtenOBP10 -RPQGPPDP---SNDEHINKYICFKECMAKELGLMDNDNNPNSDKIRDLLKHILR--GSK 133

CtenOBP6 QHRKRRSADSFFNEEEKKIAG-CLLQCVYRKMKAVNVFGFPTVEGLVSLYTEGI-----T 141

CtenOBP12 GESSGISDTTSQNREANATDG-CVVRCFLNHMHVMNVDGYPDQQRVVHGLLKEAN--GRE 222

CtenOBP3 ------------MPTTRE-AK-CLISCFVKETGL-EFDGDNVSDK--FGLLETIQKEDPD 99

CtenOBP8 ------------PPSTRE-AK-CMLACFIREAGI-EFDGNKVTDK--HGFVDIIKKEDPE 101

CtenOBP5 ------------FADDQS-LK-CYMKCIMAQMAVMDDDGMIDVE--------GAIAV--- 85

CtenOBP7 ------------FVEDEA-LM-CYYECIFKMGKL-MINGKYDLE--------SALTQVAT 93

CtenOBP13 ------------------------------MAKV-TKDGQFDED--------LALSLVSS 21

CtenOBP9 ------------FPENDENAKFKYVYCATIKENLIDKDGNMKED--------TVRLITSK 92

CtenOBP11 ------------FPESEPDAK-KYVFCVLKRIDLMDDDGNVKED--------TIKAKVPK 93

CtenOBP14 ------------FPEKEWNGK-KYLFCMMKKLGLIDDEGNLQEK--------SIRENPPV 90

CtenOBP1 ------------ITEDKA-LK-DYTFCVFEKAEFVNAEGKAQPN--------VIETKLSS 91

CtenOBP4 ------------FADDPQ-LR-NYIFCNLYKGAFLTEDGALRPQ--------IAQIKLTL 92

. .

CtenOBP2 F---EKD-TDTIFTECVKNADDNLSKYP-GQCNAHPFLFSNCYLYEVFERCPV--IRDAE 194

CtenOBP10 F---AED-ADTIFTECKAEADNKLSNYSHRQCDARSFLFSSCNLYKIFERCPENEIVDKT 189

CtenOBP6 EPEYIKGA-LQAVDACLEKAK-K---------DYA-TDLQNLQGKN----Y--------- 176

CtenOBP12 V----RDFLEDSTNECFQQ-LEGH---NLDSCEFS-TQLVMCLADRGRANCADWP----- 268

CtenOBP3 Y----YNAKLGVIKACHAE-VEK----VDDDCEYY-GKMFECEVAKAKEANIPFA----- 144

CtenOBP8 L----YNMGFEIIKACHEE-VPK----LDDECEYH-GKLFECHIKKVKETDFPPL----- 146

CtenOBP5 LPDDLKD-AADTIRKCGTK-VGKD------ACDNA-WLTHKCYYETDPEHYFLV------ 130

CtenOBP7 LPEARQELITATLKNCKDKGQGSD------KCANA-FEVAKCFYLDNPENFFLP------ 140

CtenOBP13 LPVERQEATKKTIQKCKSRGKNSD------KCVAA-FEYVKCSYFHNPK----------- 63

CtenOBP9 L--MPEDAVNKYIDTCKQN-KGSI------KYEKL-RDFFSCYLKDIIKYVAI------- 135

CtenOBP11 D--IPTEAVRQFISSCTEK-AASD------KYEKA-KNFYQCFMDNFGDAIKI------- 136

CtenOBP14 V--MPSEIIDKLIAACKQT-NGSD------KYEKA-KDVYDCILKNFNVVFT-------- 132

CtenOBP1 L--VGKETAKKLVETCTSN-VSQK---TPQKQEAS-LNIYKCIFSETKFSVI-------- 136

CtenOBP4 A--FGREKADGLLEKCKTL-SNKD------KLLYS-EELLKCIYDDTHVSII-------- 134

*

CtenOBP2 KCQKIKEDLKSGNFAHPEGQRKTVKEPNSSSA 226

CtenOBP10 KCDDIIEMLKNGRFIPLHPPPNEK-------- 213

CtenOBP6 -------------------------------- 176

CtenOBP12 ----------AGSLPFQ--Q------------ 276

CtenOBP3 ----------PFSLD----------------- 149

CtenOBP8 ----------PIAL------------------ 150

CtenOBP5 -------------------------------- 130

CtenOBP7 -------------------------------- 140

CtenOBP13 -------------------------------- 63

CtenOBP9 -------------------------------- 135

CtenOBP11 -------------------------------- 136

CtenOBP14 -------------------------------- 132

CtenOBP1 -------------------------------- 136

CtenOBP4 -------------------------------- 134

**B**

CtenCSP9 MYRAFAIPIVALLAHLRIY-----------CLAQEPPGGYYRSRYDHIDIDTVMNSKRLV 49

CtenCSP2 ---MGRPALVCLCCTFAVV-----------IVVTSAQLDKYAGRLDNINVDEVLGNDRLL 46

CtenCSP7 -MISKSIPIICAIATFVLLTDHQKVVGATPLTRNERAVDKYTTKYDNIDIDQILNNERLL 59

CtenCSP5 ---MRNVIVFCSLFLFLFP------------EDSVCAEVKYTTKWDNINVDEILKSERLL 45

CtenCSP6 ---------MKLSVFVLAV------------IVIVAAADQYTTKYDNVDVDRILSNQRVL 39

CtenCSP8 ---MSRLAIVSLVCAFVAF------------SSAVPQGDKYTVKYDNVDLEQILRNDRLL 45

CtenCSP1 ------------------------------------------------------------ 0

CtenCSP3 ---MKVFALIALFAVVVAV------------AYAAP-DGKFTTKYDNIDLEEILHNDRLL 44

CtenCSP4 ---MKGFALVALFAVVVAM------------VYAAP-DGKFTTKYDNIDLEEILHNDRLL 44

CtenCSP10 -MHLLQLLLICSLLTIALS---------------AP---QSRPMVSEEALEKTLNDRRYL 41

CtenCSP11 -MQCLT---VIVVVFVAYL---------------SP---G-------VRCQFQLHNNDYV 31

CtenCSP9 NYYGACLLGRGPCPPQGAELRRVLPEALQTNCARCTEKQKAGAFRTIKRLRKEYPDIWKE 109

CtenCSP2 TNYYKCLMDQGRCTPEAANLKKLIPTAVEKKCGNCTPNQREQLKKAITYLRAHKPDYFKD 106

CtenCSP7 KNYINCLLDKGKCTPEAEELKKHLADALATDCAKCSDVQKKQAGKILSFILQYHRDYWNQ 119

CtenCSP5 TNYFKCLMDQGRCTPDGADLKKILPDALQTKCTKCSDQQREKSKQVINHIIKEKRNWWNQ 105

CtenCSP6 TNYIKCLMEEGPCTPEGRELKKTLPDALETGCTKCNEKQKQTAEKVVRHLMKNRAKDWER 99

CtenCSP8 ENYYNCVMDKGKCTPDGQELKRNIPDALNNRCTKCSERQKAGTQRVVEYLIQNKPEWWRS 105

CtenCSP1 -------MDEGKCTPDGEELKKAIPEAIQNKCAGCSEKQKEGARKVVHFLLENKKDQFKK 53

CtenCSP3 ENYYKCLLGEGKCTPDGEELKKAIPEAIQNKCAGCNEKQKEGARKVVHFLLENKKDQFKK 104

CtenCSP4 ENYYKCLLGEGKCTPDGEELKKAIPEAIQNKCAGCNEKQKEGARKVVHFLLENKKDQFKK 104

CtenCSP10 MRQLKCALGEAPCDSVGRRLKSLAPLVIRGSCPQCTPTEMRQIQKVLAHVQKNYPREWGR 101

CtenCSP11 EKQLLCALDRGPCDFLGHQIKQVLPNVIGQNCANCSPRESAYAARIADFVQKNYPAVWVQ 91

: .. * . :: .: * *. : : : :

CtenCSP9 LVEEYDPTEKYVRRFEETMLKKKPYLTSSSFPNRASFDTNDVQNPSPSPSNVSPVTLTTI 169

CtenCSP2 LVAKYDPNETWKGLYE-------------------------------------------- 122

CtenCSP7 LLDKYDPSGNFRKKYELEEDKKK------------------------------------- 142

CtenCSP5 LENKYDPESKYKTIYKQEIADLGITLD--------------------------------- 132

CtenCSP6 LSAKYDPNGEYKKRLEKQYRAQ-------------------------------------- 121

CtenCSP8 LETKYDPSGNYRRAYGPELAQRGIKV---------------------------------- 131

CtenCSP1 LVEKYDPNGSYREANKELLEKEGIHF---------------------------------- 79

CtenCSP3 LVEKYDPDGSYRAANKDLLEKEGIHF---------------------------------- 130

CtenCSP4 LVEKYDPDGSYRAANKDLLEKEGIHF---------------------------------- 130

CtenCSP10 ILQQYSSG---------------------------------------------------- 109

CtenCSP11 LVQKYRASG--------------------------------------------------- 100

: :*

CtenCSP9 RDVITTPASSNKIGAAAITTTTSTTSTTTTTTTTPNTASLSTTKPKTTNSFTTLSSKRGT 229

CtenCSP2 ------------------------------------------------------------ 122

CtenCSP7 ------------------------------------------------------------ 142

CtenCSP5 ------------------------------------------------------------ 132

CtenCSP6 ------------------------------------------------------------ 121

CtenCSP8 ------------------------------------------------------------ 131

CtenCSP1 ------------------------------------------------------------ 79

CtenCSP3 ------------------------------------------------------------ 130

CtenCSP4 ------------------------------------------------------------ 130

CtenCSP10 ------------------------------------------------------------ 109

CtenCSP11 ------------------------------------------------------------ 100

CtenCSP9 PRPASSAGRQTTIKTDTRSTSSGNAFEPLPLFVPQTFSESEPPTKVTTNTXETGLSRLGQ 289

CtenCSP2 ------------------------------------------------------------ 122

CtenCSP7 ------------------------------------------------------------ 142

CtenCSP5 ------------------------------------------------------------ 132

CtenCSP6 ------------------------------------------------------------ 121

CtenCSP8 ------------------------------------------------------------ 131

CtenCSP1 ------------------------------------------------------------ 79

CtenCSP3 ------------------------------------------------------------ 130

CtenCSP4 ------------------------------------------------------------ 130

CtenCSP10 ------------------------------------------------------------ 109

CtenCSP11 ------------------------------------------------------------ 100

CtenCSP9 ELGRLSFQLCKLLLGSNSEFCYAKHNVRRRRKTTYWSTLCPFAFN 334

CtenCSP2 --------------------------------------------- 122

CtenCSP7 --------------------------------------------- 142

CtenCSP5 --------------------------------------------- 132

CtenCSP6 --------------------------------------------- 121

CtenCSP8 --------------------------------------------- 131

CtenCSP1 --------------------------------------------- 79

CtenCSP3 --------------------------------------------- 130

CtenCSP4 --------------------------------------------- 130

CtenCSP10 --------------------------------------------- 109

CtenCSP11 --------------------------------------------- 100
